# Acceptance and perceived value of non-invasive malaria diagnostic tests in malaria-endemic countries

**DOI:** 10.1186/s12936-021-03911-y

**Published:** 2021-09-24

**Authors:** Ewurama Dedea Ampadu Owusu, Ana Campillo, Jennifer Daily, Xavier C. Ding

**Affiliations:** 1 FIND, Geneva, Switzerland; 2 Department of Medical Laboratory Sciences, School of Biomedical and Allied Health Sciences, College of Health Sciences, University of Ghana, Accra, Ghana

**Keywords:** Malaria, Rapid diagnostic test, User acceptability, Non-invasive diagnostic test, Saliva-based test, Urine-based test, Use-case scenarios

## Abstract

**Background:**

The diagnosis of malaria, using microscopy or rapid diagnostic tests (RDTs), requires the collection of capillary blood. This procedure is relatively simple to perform but invasive and poses potential risks to patients and health workers, arising from the manipulation of potentially infectious bodily fluids. Less or non-invasive diagnostic tests, based on urine, saliva or requiring no sampling, have the potential to generate less discomfort for the patient and to offer simpler and less risky testing procedures that could be safely performed by untrained staff or even self-performed. To explore the potential acceptance and perceived value of such non-invasive tests, an online, international survey was conducted to gather feedback from National Malaria Control Programme (NMCP) representatives.

**Methods:**

An online survey comprising nineteen questions, available in English, French or Spanish, was emailed to 300 individuals who work with NMCPs in malaria-endemic countries. Answers were collected between November and December 2017; responses were qualitatively analysed to identify key themes and trends and quantitatively analysed to determine average values stratified by region.

**Results:**

Responses were received from 70 individuals, from 33 countries. Approximately half of the respondents (52 %) considered current blood-based tests for malaria to be minimally invasive and non-problematic in their setting. For these participants, non-invasive tests would only be of interest if they brought additional performance improvements, as compared with the performance of microscopy and RDTs. Most respondents were of the view that saliva-based (80 %) and urine-based (66 %) tests would be more readily acceptable among children than blood-based tests. Potential use-case scenarios of interest for both saliva- and urine-based tests were ease-of-testing by community health workers, additional surveillance, self-testing, and outbreak investigation. Many respondents (41 %) thought that if saliva-based tests retailed at <$0.50 per unit they could largely replace conventional RDTs, whereas only 25 % of respondents thought a similarly priced urine-based test would do so.

**Conclusions:**

Although limited to NMCP stakeholders, this survey indicated that current tests for malaria, based on capillary blood, are generally perceived to be minimally invasive and non-problematic. Non-invasive tests, especially if saliva-based, would be welcome if they could match or out-perform the price and performance of current blood-based tests.

**Supplementary Information:**

The online version contains supplementary material available at 10.1186/s12936-021-03911-y.

## Background

Malaria is associated with non-specific symptoms; therefore, suspected cases must be investigated using a parasitological diagnostic test. Since 2010, the World Health Organization (WHO) has recommended quality-assured light microscopy or rapid diagnostic tests (RDTs) as acceptable means of carrying out this diagnosis [1]. Whereas light microscopy is technically complex and requires a highly trained laboratory technician, RDTs are affordable, easy-to-use, robust, and generally provide acceptable performance for clinical case investigation. RDTs have, therefore, been widely adopted as the primary diagnostic procedure for malaria, especially in countries where *P*. *falciparum* malaria is endemic, such as in sub-Saharan Africa (SSA), where an estimated 348 million RDT units were reported to have been sold by manufacturers in 2019 [2]. From 2005 to 11 to 2015-19, the proportion of febrile children aged under 5 seeking care who received a diagnosis in SSA increased from 15.4 to 37.7 %, essentially driven by this increase in RDT usage [2].

Both light microscopy and RDTs require the collection of a capillary blood sample, typically obtained from a finger or heel prick. This procedure is minimally invasive but is, nevertheless, associated with transient pain at the site of pricking. It is usually performed using a disposable, single-use lancet to minimize risk. Yet, any manipulation of blood samples carries an inherent infection risk for both patients and healthcare workers. Capillary sampling is often considered to be a simple procedure, despite requiring no less than eleven distinct steps when performed according to WHO guidelines on best practice in phlebotomy [3]. It is not clear if these recommendations, including proper waste disposal, can always be fully applied when testing for malaria, especially at the most decentralized level of health systems, where RDTs are most often performed and where medical infrastructure is typically lacking. The perceived low level of risk associated with capillary sampling might itself represent an additional risk factor, since RDT users or microscopists might not necessarily recognize the need to follow strict guidelines when performing this procedure. Given that RDTs are essentially all blood-based and, therefore, require invasive blood-drawing techniques, this may also influence compliance among patients where there is cultural reluctance involved in giving blood, if repeated sampling becomes necessary or when asymptomatic individuals are being tested [4, 5].

The use of alternative, non-invasive sample types that could avoid pricking discomfort, minimize infectious risks and simplify waste disposal might, therefore, be of interest for diagnosing clinical cases of malaria. Avoiding invasive sample collection might also facilitate interventions based on the active screening of asymptomatic individuals who, by definition, feel healthy and might be less inclined to provide a blood sample requiring pricking.

Additionally, it is increasingly recognized that universal access to anti-malarial interventions is necessary. Therefore, a non-invasive diagnostic test for malaria might also represent a way to further increase the proportion of febrile individual seeking care in both public and private health sectors benefiting from a malaria diagnosis, as such a test would be easier to administer and could overcome any cultural or comfort sensitivities related to pricking and blood collection.

Non-invasive tests for malaria can involve the detection of plasmodial parasite antigens or DNA in samples other than blood, such as saliva, urine or buccal mucosa [6]. There are also diagnostic tests that do not require sample collection, for example those that use skin volatiles as predictors of infection status or the use of harmless laser pulses to the skin to generate then detect vapour nano-bubbles in malaria parasites (nano-bubble transdermal detection) [7, 8]. Many of these tests are still in the exploratory stages of development; however, the development of saliva- and urine-based tests is well advanced, with pilot studies and clinical trials for urine-based RDTs having been conducted in Nigeria and India, among other countries and with R&D programmes focusing on saliva markers for the detection of malaria [9–11]. Although urine- and saliva-based tests may be promising complements to blood-based tests, their acceptance and desirability may largely depend on how they are technically and culturally perceived. This, together with the performance and cost of a test, will have a direct impact on the usefulness of such non-invasive tests. Consideration of beneficiaries and users at different levels, such as the individuals being tested, the health care providers performing the tests, NMCPs and donors, will be necessary because of the varied stakeholders involved in effective diagnostic coverage.

The aim of this study was to identify and outline the acceptability and perceived value of non-invasive RDTs for malaria, with a particular focus on urine- and saliva-based tests, since these are at the most advanced stage of development. This work sought to establish the level of acceptance of saliva- and urine-based tests and to identify the preferred product characteristics and use-case scenarios for these tests in low- and middle-income countries (LMICs) through a large-scale survey targeting NMCP collaborators.

## Methods

### Data collection

An online survey form was created using Google Forms; this was administered in English (Additional file 1), with translations in French (Additional file 2) and Spanish (Additional file 3) also made available, as appropriate, to individuals working in local institutions in malaria-endemic countries. The contact details of these individuals were obtained from a database maintained by the Foundation for Innovative New Diagnostics (FIND). The survey was conducted from mid-November to early December 2017 and comprised nineteen questions, including questions relating to major goals and barriers in terms of diagnostics, use-case scenarios for non-invasive tests, the need for and potential impact of non-invasive tests, the acceptability of non-invasive samples, and preferred product characteristics (such as costs and sample types).

### Analysis

The data obtained were translated into uniform English language. Basic, country level epidemiological data from the 2017 World Malaria Report and the Malaria Atlas Project (MAP) were incorporated [12]. Open-ended questions were qualitatively reviewed to identify key themes. The data were aggregated into tables and relevant charts were generated using Microsoft Excel (Version 2013). Endemic countries were classified as “control countries” and “elimination countries” according to the UCSF Global Health Group’s Malaria Elimination Initiative classification.

## Results

### Demographics of the survey

**Fig. 1:**
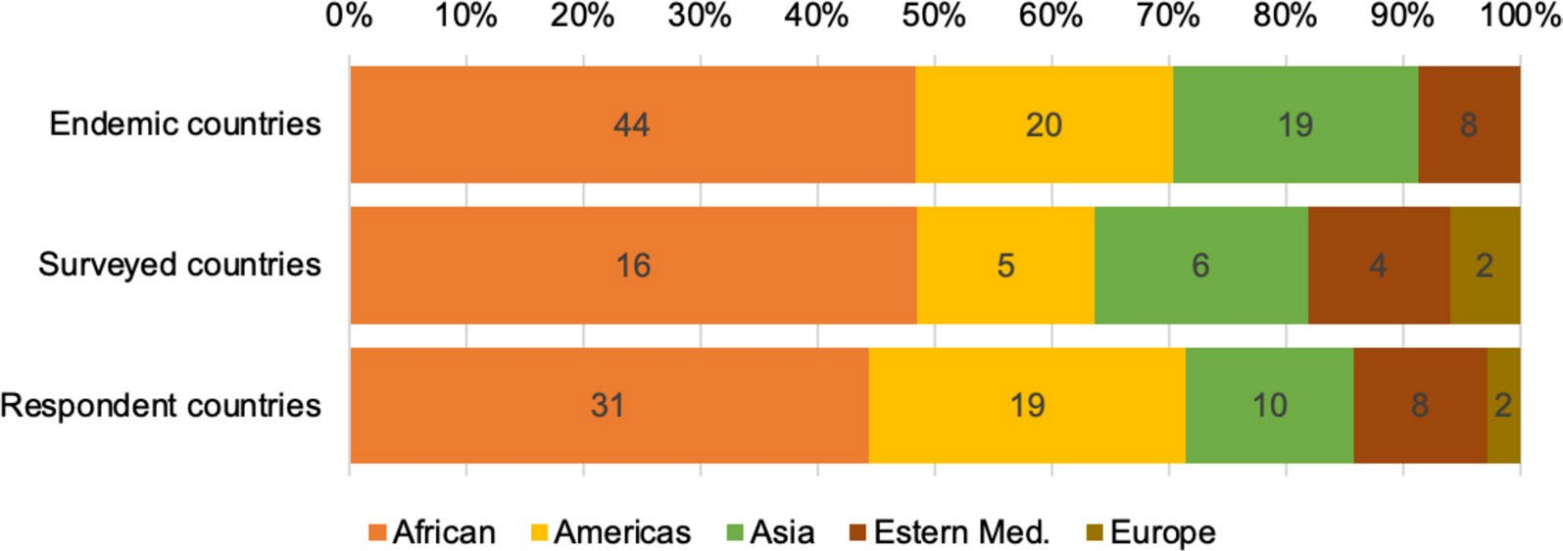
Distribution of surveyed countries and respondent countries as compared to malaria endemic countries according to WHO regions

The questionnaire was pre-tested with ten participants, selected at random from the pre-established contact list. However, no further adjustments were identified during this pre-test; therefore, these participants’ responses were included in the final analysis and the unchanged questionnaire was deployed. A total of 300 questionnaires were sent out, of which 73 (24.3 %) were completed and returned. Of the returned questionnaires, three were excluded from the analysis because the respondents did not work on a full-time basis in malaria-endemic countries. The survey respondents were based in 33 (36.3 %) of the 91 WHO-recognized malaria-endemic countries around the world (Fig. 1). Responses from two countries in Europe where malaria was recently eliminated (Armenia and Georgia) were also included.

### Perceptions on current issues related to malaria testing

**Fig. 2:**
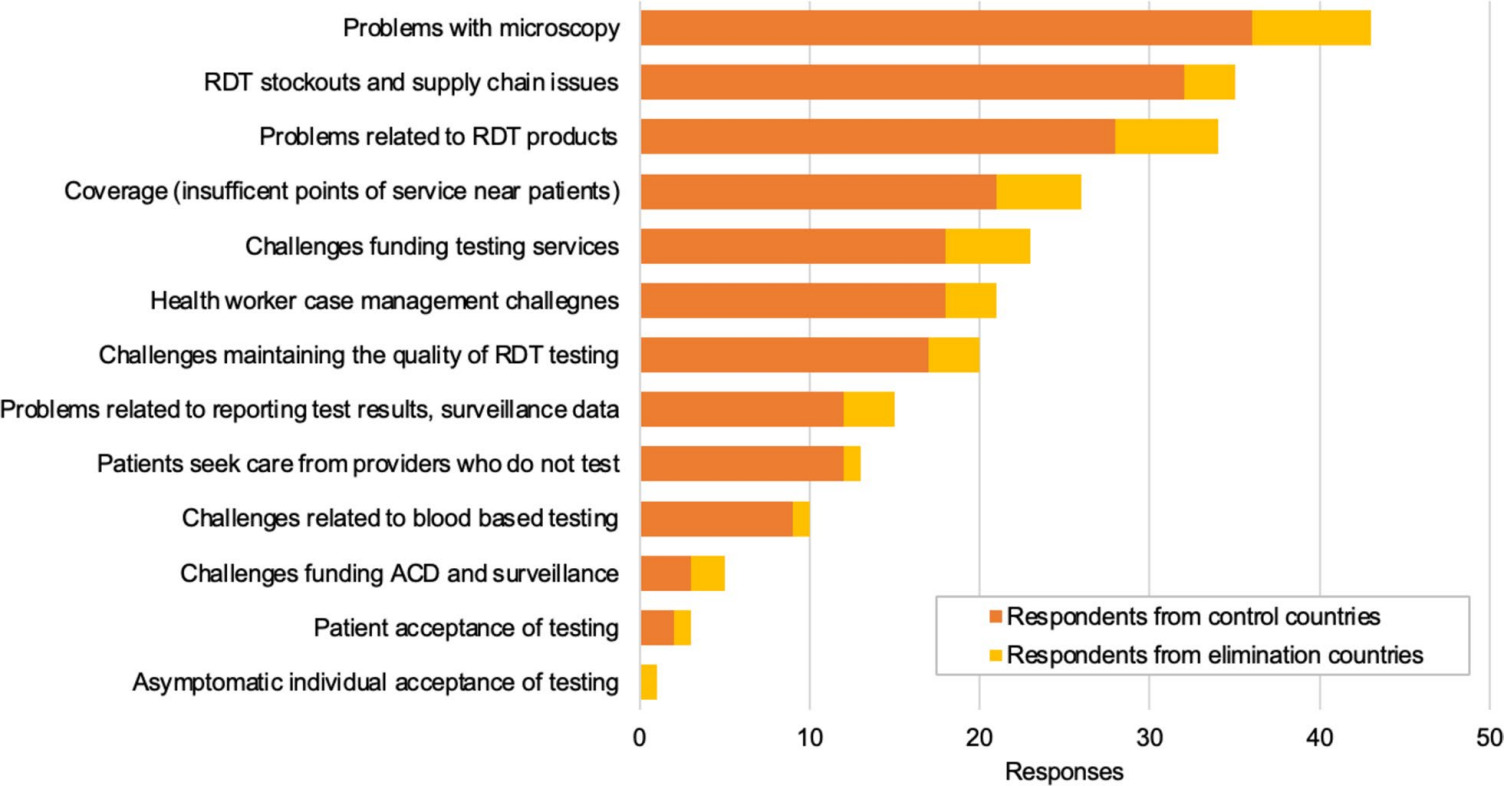
Perceived Programme challenges in achieving its goal related to testing. Respondents were asked to select up to three items from a list of answers and were given the opportunity to select and define an unlisted answer (“other”). “Problem with microscopy” includes any type of problem such as training staff, quality, supply chain, etc. “Problems related to RDT products” includes ability to pick up asymptomatic infections, HRP2 deletions, speciation, persistence of antigens, etc. “Asymptomatic individual acceptance of testing” was not listed and defined by a respondent

When interrogated about what could “get in the way of the [national malaria] program reaching its goals around testing”, the top three potential concerns selected by respondents from a list of suggestions were problems with microscopy (including any type of problem, such as training staff, quality, supply chain), RDT stock outs and supply chain issues, as well as problems related to RDT products (including inability to detect asymptomatic infections, HRP2 deletions, speciation, persistence of antigens) (Fig. 2). “Challenges related to blood-based testing” was amongst the four potential concerns least cited (selected by 10 out 70 respondents).

### The need for and potential impact of non-invasive tests

**Fig. 3:**
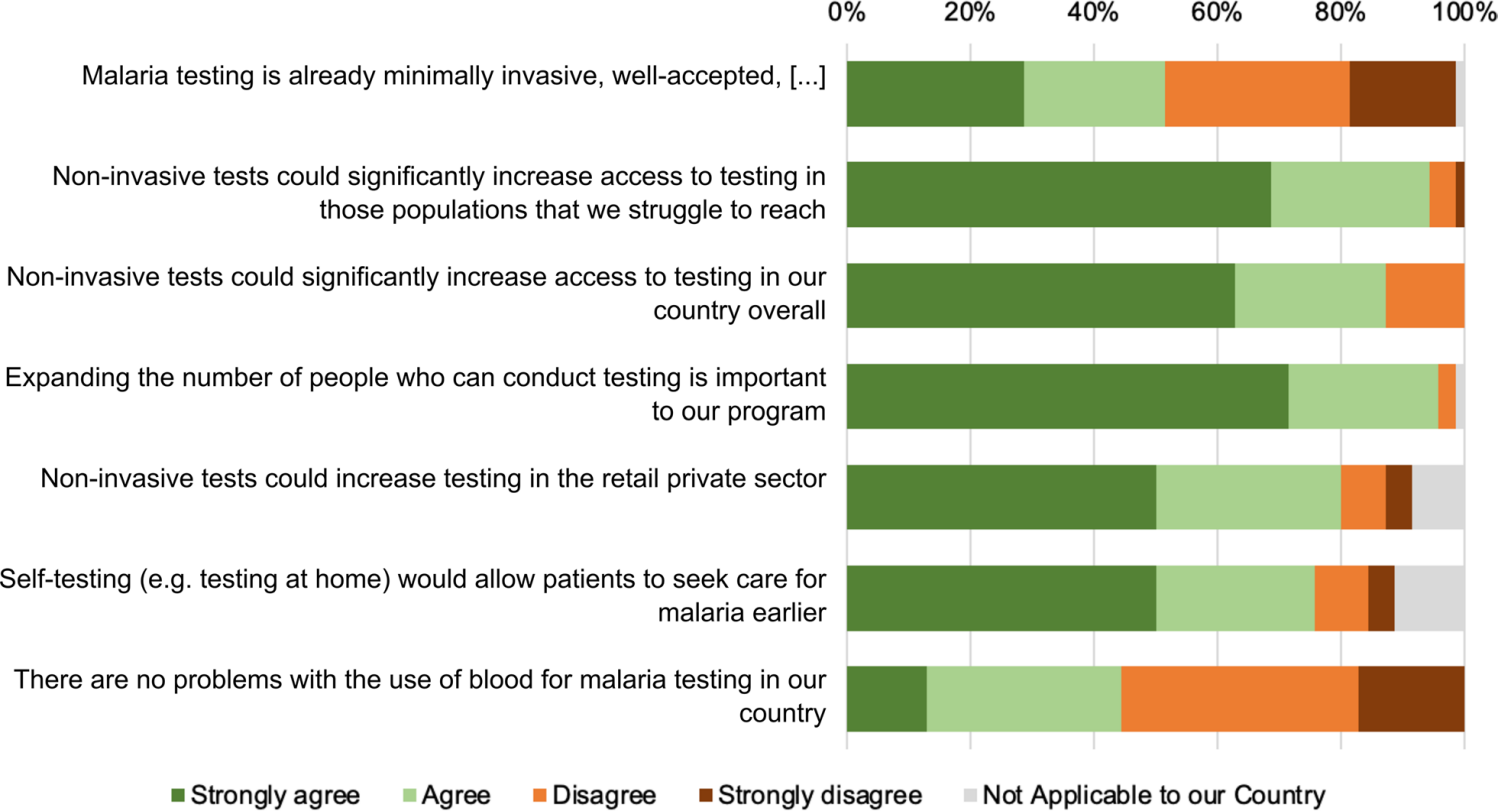
The need for and potential impact of non-invasive tests. Respondents were asked to indicate if the “strongly agree”, “agree”, “disagree”, “strongly disagree” with seven different statements about the potential impact of non-invasive malaria tests. The complete first statement is “Malaria testing is already minimally invasive, well-accepted, and common in our country, there is no need to change to non-invasive tests unless new tests have other advantages (including significantly improved performance, turn-around time)”

The survey indicated that just over half of the respondents (36/70, 51 %) agreed that the current approach to malaria testing is minimally invasive and that unless any new tests have other advantages, there is no need for change (Fig. 3). However, a majority (39/70, 56 %) disagreed that there were no problems at all with the use of blood for malaria testing. With regards to the potential public health benefits of non-invasive tests, the majority of respondents agreed that non-invasive tests could significantly facilitate malaria diagnosis in areas that are difficult to reach (66/70, 94 %) and overall access to testing in their country (61/70, 87 %). A majority of respondents also believed that non-invasive tests could increase testing in the retail private sector (56/70, 80 %) and allow patients to seek care for malaria earlier, following potential self-testing (53/70, 76 %).

### Acceptance of non-invasive samples and preferred product characteristics

In predicting how readily acceptable saliva-based tests would be, a vast majority of the respondents (56/70, 80 %) held the view that these tests would be most readily accepted among children (Table 1). Similarly, for urine-based tests, children were thought most likely to readily accept the tests (46/70, 66 %), although at a lower percentage than saliva-based tests. Despite 61 % of respondents (43/70) stating that there were no major barriers in relation to cultural or religious beliefs or reasons of tradition, when it came to taking blood samples, some respondents commented on the importance of communication and the sensitivities of some communities to new methods of sampling.

**Table 1:** Predicted acceptance of urine and saliva-based tests by categories of users (n = 70)

Group/category	Readily accept	Hesitant initially, eventually accept	Unlikely to use/prefer blood	Not applicable to our country
Predicted acceptance for saliva based tests				
Children	80 %	10 %	6 %	4 %
Adults	50 %	39 %	9 %	3 %
Migrant/mobile populations	66 %	23 %	7 %	4 %
Indigenous people	51 %	34 %	7 %	7 %
Remote/rural communities	59 %	30 %	7 %	4 %
Populations being tested as part of a survey (mostly asymptomatic)	67 %	23 %	7 %	3 %
Professional health care workers (e.g. doctors, nurses)	57 %	29 %	11 %	3 %
Community health workers	63 %	30 %	6 %	1 %
Retailers (drug stores, pharmacists)	49 %	21 %	10 %	20 %
Predicted acceptance for urine-based tests				
Children	66 %	26 %	4 %	4 %
Adults	49 %	36 %	13 %	3 %
Pregnant women	65 %	24 %	7 %	4 %
Migrant/mobile populations	51 %	30 %	13 %	6 %
Indigenous people	40 %	43 %	10 %	7 %
Remote/rural communities	50 %	37 %	10 %	3 %
Populations being tested as part of a survey (mostly asymptomatic)	43 %	43 %	9 %	6 %
Professional health care workers (e.g. doctors, nurses)	43 %	40 %	14 %	3 %
Community health workers	50 %	41 %	6 %	3 %
Retailers (drug stores, pharmacists)	30 %	26 %	19 %	24 %

### Use-case scenarios for non-invasive tests by NMCPs and local institutions in low- and middle-income countries

**Fig. 4:**
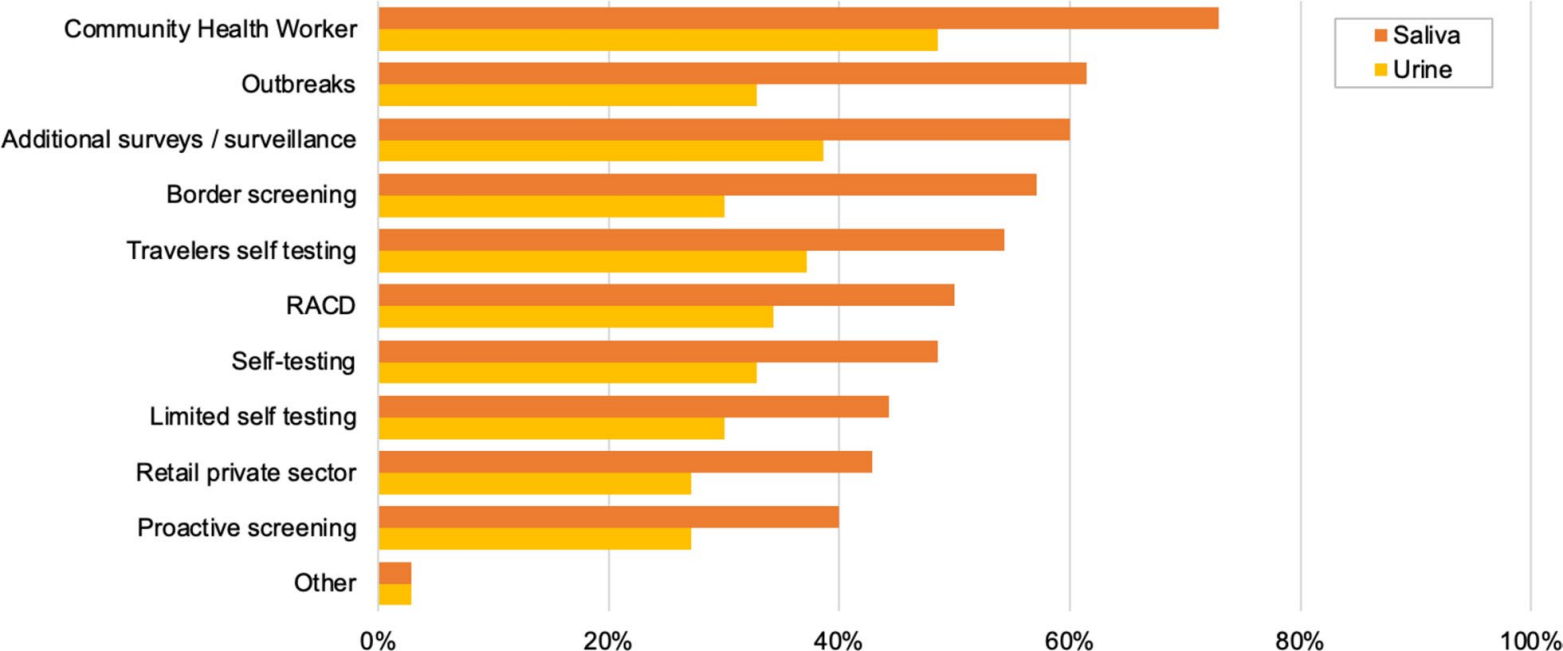
Activities that National Malaria Control Programmes would consider starting if non-invasive malaria diagnostic tests were available. RACD is reactive case detection

A potential attractiveness of new malaria tests not requiring blood collection is the possibility to use them for activities not readily compatible with the practicalities of blood collection. When questioned about what such activities could be, the top-five new activities or initiatives that malaria control programmes would consider performing if saliva-based tests were available were the introduction or reinforcement of community health workers (CHWs) (51/65), outbreak investigation (43/65), additional surveillance (42/65), border screening (40/65) and traveller self-testing (30/65), in that order (Fig. 4). The top-five new activities or initiatives if urine-based tests were available were the introduction or reinforcement of CHWs (34/45), additional surveillance (27/45), traveller self-testing (26/45), reactive case detection (24/45), and outbreak investigation (23/45).

Survey respondents’ opinions on the key product characteristics that should be prioritized are shown in Table 2. The top preferences were for product stability at 40 °C, a product capable of detecting sub-microscopic infections, and a product that is easy to use for untrained lay people.

**Table 2:** Key product characteristics to be prioritized

Product characteristic	Priority	Percentage*	
*Plasmodium spp.* detection			
	Pan/*P*. *falciparum*	40.0 %	
	*P*. *falciparum*/*P*. *vivax*	46.0 %	
Price per purpose		**< $0.50**	**> $0.50**
Saliva-based	Largely replace both conventional RDTs and microscopy	32.00 %	0.76 %
	Largely replace conventional RDTs	41.00 %	17.42 %
Urine-based	Largely replace both conventional RDTs and microscopy	16.90 %	5.16 %
	Largely replace conventional RDTs	25.35 %	15.02 %
Stability at 40 °C			
	Absolutely essential	76.0 %	
	Probably not needed	1.0 %	
Ability to detect sub–microscopic infections			
	Absolutely essential	74.0 %	
	Probably not needed	3.0 %	
Can be used by untrained lay people			
	Absolutely essential	74.0 %	
	Probably not needed	1.0 %	
Shelf-life			
24 month shelf–life	Absolutely essential	66.0 %	
	Probably not needed	1.0 %	
18 month shelf–life	Absolutely essential	43.0 %	
	Probably not needed	14.0 %	
Time to results: faster than current RDTs			
	Absolutely essential	46.0 %	
	Probably not needed	9.0 %	
Quantitative results			
	Absolutely essential	31.0 %	
	Probably not needed	10.0 %	

Bold values “<0.50” and “>0.50” represent categories for which answers are given below (for instance 32% of respondant think that saliva based test will “largely replace conventional RDTs and microscopy”

*Percentages do not add up to 100 because only the two most relevant options are presented here

### Perceived attractiveness and potential of adoption of non-invasive tests for malaria

**Fig. 5:**
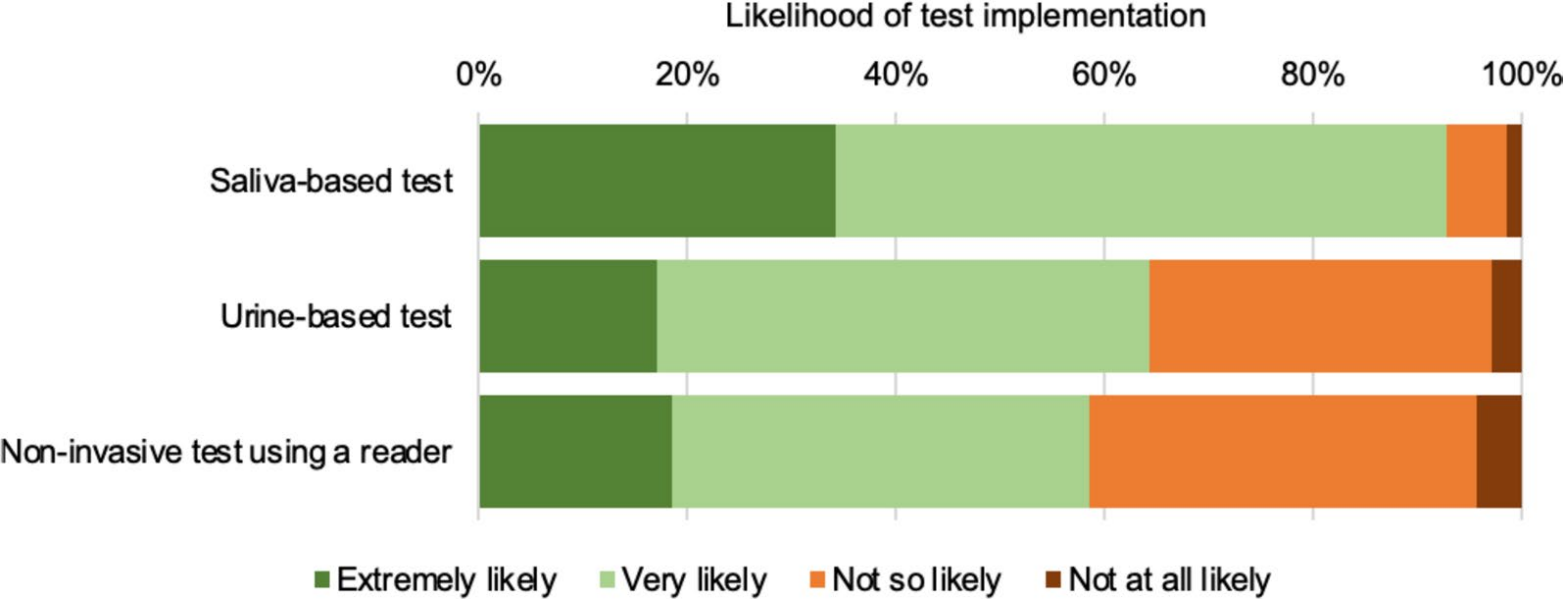
Likelihood of test implementation according to the required sample type

Assuming they would perform equally well, respondents were asked to indicate which of blood, saliva or urine would be their first-choice sample type for a malaria test. Saliva was mentioned by 79 % of the respondents, blood by 13 %, and saliva by 8 %. Irrespective of price considerations, 93 % of respondents (65/70) were of the opinion that a saliva-based test would be implemented if available (“extremely likely” and “very likely”) (Fig. 5). The perceived likelihood of implementation for urine-based tests and for non-invasive test using a reader was less positive, with only 64 and 59 % of respondents seeing it as extremely or very likely to happen. The main reasons cited for not implementing urine-based test where the operational difficulties in obtaining the sample (24 %, 6/25) and satisfaction with current blood-based tests (24 %, 6/25).

**Fig. 6:**
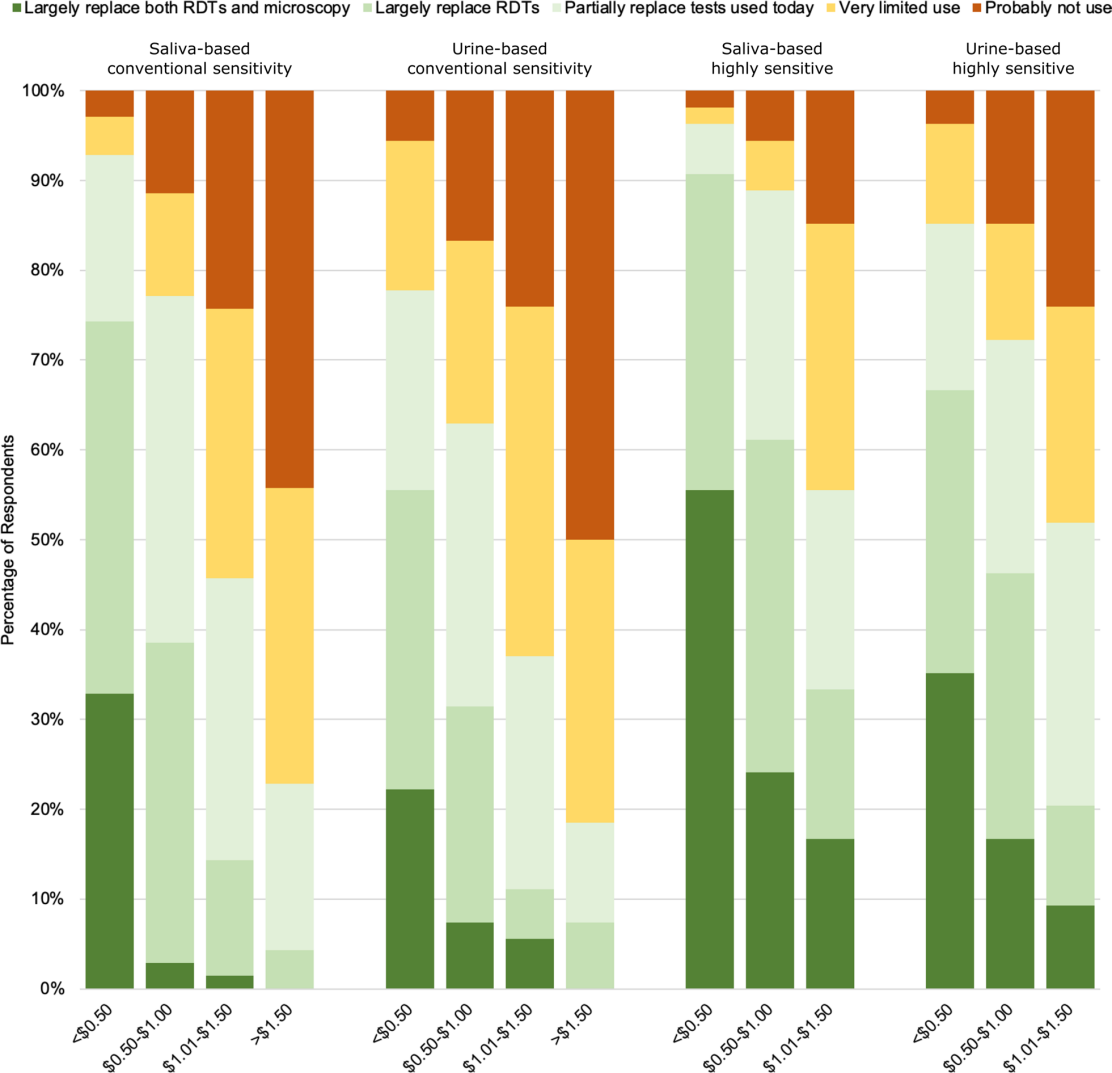
Perceived value proposition of conventional and “highly sensitive” tests based on saliva and urine

Price is a key determinant of the actual and perceived value of health commodities. To assess the willingness to pay for saliva and urine-based tests, survey participants were asked to indicate how their Programme might use this type of tests according different potential price categories (Fig. 6). Respondents indicated a clear preference for more sensitive tests based on saliva and associated with a price below 50 US cents per unit, with 98 % indicating that such a test could at least partially replace tests used today. Results showed a high price sensitivity, with close to 50 % of the respondents indicating that any test between 1.00 and 1.50 US dollars per unit would have very limited to no use, regardless of its sensitivity and sample type.

## Discussion

The opinions of individuals working for local malaria control institutions in LMICs, but not speaking in their official, formal capacities, and gathered through an online survey are reported here. The analysis of the survey data outlines the perception of non-invasive malaria test utility and their anticipated added-value. In addition, information relating to the level of acceptance of non-invasive sampling, preferred product characteristics, and use-case scenarios in LMICs have been obtained. The overall response rate to the survey was low, at 24.3 %. Nonetheless, about one-third of the 91 WHO-recognized malaria-endemic countries were represented, covering all WHO regions and providing good baseline results for developing further insights.

There was consensus among survey respondents that non-invasive tests could increase people’s access to malaria testing, thus providing a potential public health benefit. Hard-to-reach groups, such as migrants, refugees and remote communities are specific population groups that respondents suggested could benefit from non-invasive tests. Improved access to diagnostics has been associated with reductions in private household costs such as the consultation fees, drugs, transport and food during each episode and the overall burden of malaria [2]. A further direct benefit of improved diagnostics is a reduction in unnecessary treatment. This may have advantages both in impeding the proliferation of drug-resistant parasites and in saving lives.

The type of sample preferred varied by region; while the Americas, African and Mediterranean regions preferred saliva-based tests, respondents from Asia were open to both urine- and saliva-based tests. This may be more attributable to individuals’ reservations about handling a particular sample type, such as urine, rather than any widely held cultural beliefs, as was explained by some respondents. Thus, respondents emphasized the importance of sensitivity and effective communication if the introduction of new sample types is to be successful.

With regards to use-case scenarios, CHWs, self-testing among travellers, outbreak monitoring, and additional surveillance were commonly identified potential advantages for both urine- and saliva-based tests. Less invasive diagnostic tests will require little to no technical skill and could, therefore, be used by anyone as a home test, during outbreaks, or by travellers. CHWs will also require very little training, and malaria surveillance would be improved. Such an increase in diagnostic testing could dramatically improve malaria control, beyond that observed in recent years. Recently, the expansion and strengthening of malaria diagnostics has been demonstrated to make a major contribution to the progress made in malaria control [2].

Most respondents considered blood-based tests for malaria to be minimally invasive, which may reflect perceptions around finger-pricking for blood-based testing more broadly. For example, in a survey of pregnant women in Ghana, participants expressed more positive feelings towards intermittent screening for malaria, which involved finger-pricking for blood testing, compared with their feelings towards intermittent preventive treatment for malaria, despite the latter not requiring blood testing [13]. In a survey of potential lay users of HIV self-test kits in South Africa, respondents reported the kits as being easy to use, regardless of whether administered via oral swabs or finger pricks, although some respondents noted that they experienced pain and others reported difficulty in using the lancets supplied [14].

One of the key product characteristics identified by respondents included product stability at 40 °C; this is consistent with the occurrence of malaria in tropical areas with high temperatures and with the typical temperature stability offered by current blood-based RDTs. Many survey respondents also stated a preference for urine- or saliva-based malaria RDTs that could detect sub-microscopic infections and could be used by untrained lay people. The limit of detection of currently available blood-based malaria RDTs and microscopy is 100–200 parasites/µL [15, 16]. Sub-microscopic infections are usually only detectable using molecular techniques, such as PCR, which is expensive and requires skilled experts and a well-equipped laboratory [16], or simplified nucleic acid amplification-based techniques, such as loop-mediated isothermal amplification (LAMP), which allows high-throughput DNA amplification with minimal laboratory infrastructure [17]. Additionally, a malaria RDT, with a reported 10-fold increased analytical sensitivity, has been made available recently with the objective to facilitate the detection of low-density asymptomatic malaria infections [15]. This improved RDT has been shown to have generally better clinical sensitivity than conventional RDTs, although the degree of improvement varies across settings [15, 18, 19]. Despite these improvements in the detection of low levels of parasites, they remain blood-based, invasive tests. Thus, a non-invasive RDT that possessed these product characteristics would be a major improvement on existing malaria diagnostics.

HRP2 and pLDH can be found saliva and urine at levels compatible with detection by current lateral flow assays, yet repurposing blood-based RDT detecting HRP2 and pLDH for use with urine or saliva has shown varying levels of performance, but generally below those achieved when using the corresponding test with the intended blood sample [20–23]. As highlighted by a recent review of the literature, the deployment of currently available point-of-care test using non-invasive samples is currently not feasible due to limited performance as compared to blood samples [24]. On the other hand, the performance of a HRP2-based test developed specifically to be used with urine samples has been reported to be comparable with that of blood-based RDTs, suggesting that achieving the desired performance characteristics highlighted in this report is, on a technical level, feasible [25]. Prospects for future highly sensitive saliva-based test also exist based on new promising biomarkers in development [11].

## Study limitations

This study has provided novel baseline insights into the perceived acceptance and potential impact of non-invasive diagnostics in LMICs, but shows a number of limitation. First, only about a quarter of the individuals invited to participate responded to the survey. The pool of respondents might, therefore, be biased for individuals with a strong opinion about non-invasive tests and might not be fully representative of the general perception on this type of tests. Also, even though the survey is representative on a country level, there exists regional over-representation in the Americas and under-representation in Asia. Most questions were closed-ended or semi closed-ended, providing a list of predefined answers to select from which can potentially influence the respondents. This survey was also limited to individuals working in local institutions in malaria-endemic countries and did not include the actual end-users of malaria tests, such as laboratory technician and community health works as well as patients undergoing malaria testing, who might have significantly different opinions on this topic.

## Conclusions

Current blood-based tests appear largely adequate to survey respondents. Non-invasive urine- or saliva-based tests may be acceptable to end-users in low- and middle-income countries but would need to at least match, or ideally outperform, current blood-based test to become a preferred solution over existing tests. Highlighted important characteristics for such tests include the ability to detect sub-microscopic infections, to be used by untrained lay people, stability at temperatures up to 40 °C, as well as low cost. Current efforts with blood-based malaria rapid diagnostic tests are aimed at overcoming specific limitations such as *pfhrp2* deletion or the relatively limited sensitivity of RDT for *Plasmodium vivax*. However, non-invasive tests that are saliva-based should also be considered, if the full potential offered by diagnostics is to be reached in the context of controlling malaria.

## Supplementary Information

**Additional file 1. **Structured questionnaire in English.

**Additional file 2. **Structured questionnaire in French.

**Additional file 3. **Structured questionnaire in Spanish.

## Data Availability

The datasets used and/or analysed during the current study are available from the corresponding author upon reasonable request.
