# Supplementary material for: Acceptance and perceived value of non-invasive malaria diagnostic tests in malaria-endemic countries
Source: Malar J. 2021 Sep 24;20:379. doi: 10.1186/s12936-021-03911-y (PMC8461884; doi:10.1186/s12936-021-03911-y)
Supplement: Supplementary file 1 — Additional file 1. Structured questionnaire in English. [file 12936_2021_3911_MOESM1_ESM.pdf]

Non-invasive malaria test survey

* Required

FIND Because diagnosis matters

Thank you for participating in this survey. Please click on "NEXT" to start the interview

INTRODUCTION AND INSTRUCTIONS

The Bill and Melinda Gates Foundation is considering supporting the development of noninvasive malaria tests. These would be similar to malaria RDTs, but would use either saliva or urine as a sample, instead of blood. Before investing significantly in development of these tests, the Foundation has asked us to help them understand the market for non-invasive tests, as well as to estimate any potential impact that non-invasive tests might have on global malaria control and elimination targets.

In connection with this, we would like your feedback on several areas, which we have grouped into four sections:

I) Malaria program priorities and challenges related to testing;
II) Potential use scenarios / key populations for non-invasive tests;
III) Feedback on adoption and pricing; and
IV) Preferences regarding several key product characteristics.

When answering these questions, please consider your country and national program's experience. Your answers will be used in aggregate, and we will not be evaluating individual responses. As such, please be as honest as possible – there are no right or wrong answers.
We recognize that it is difficult to provide views on a product that does not yet exist, and that you may not know the answer to some of the survey questions; we are looking for informed assumptions and predictions, based on your experience.

We anticipate that the survey will take 20 minutes to complete. Thank you in advance for your help with this important study!

DEMOGRAPHICS

The survey is anonymous, but we would like to be able to analyze the data by region and to look at the responses by institution (e.g. national malaria program responses).

1. **1. In which country do you currently work? (If you work in different countries, please select the one you spend most time working in)** *

Mark only one oval.

- Afghanistan
- Albania
- Algeria
- American Samoa
- Andorra
- Angola
- Anguilla
- Antigua & Barbuda
- Argentina
- Armenia
- Aruba
- Australia
- Austria
- Azerbaijan
- Bahamas, The
- Bahrain
- Bangladesh
- Barbados
- Belarus
- Belgium
- Belize
- Benin
- Bermuda
- Bhutan
- Bolivia
- Bosnia & Herzegovina
- Botswana
- Brazil
- British Virgin Is.
- Brunei
- Bulgaria
- Burkina Faso
- Burma
- Burundi
- Cambodia
- Cameroon
- Canada
- Cape Verde
- Cayman Islands
- Central African Rep.

- Chad
- Chile
- China
- Colombia
- Comoros
- Congo, Dem. Rep.
- Congo, Repub. of the
- Cook Islands
- Costa Rica
- Cote d'Ivoire
- Croatia
- Cuba
- Cyprus
- Czech Republic
- Denmark
- Djibouti
- Dominica
- Dominican Republic
- East Timor
- Ecuador
- Egypt
- El Salvador
- Equatorial Guinea
- Eritrea
- Estonia
- Ethiopia
- Faroe Islands
- Fiji
- Finland
- France
- French Guiana
- French Polynesia
- Gabon
- Gambia, The
- Gaza Strip
- Georgia
- Germany
- Ghana
- Gibraltar
- Greece
- Greenland
- Grenada

- Guadeloupe
- Guam
- Guatemala
- Guernsey
- Guinea
- Guinea-Bissau
- Guyana
- Haiti
- Honduras
- Hong Kong
- Hungary
- Iceland
- India
- Indonesia
- Iran
- Iraq
- Ireland
- Isle of Man
- Israel
- Italy
- Jamaica
- Japan
- Jersey
- Jordan
- Kazakhstan
- Kenya
- Kiribati
- Korea, North
- Korea, South
- Kuwait
- Kyrgyzstan
- Laos
- Latvia
- Lebanon
- Lesotho
- Liberia
- Libya
- Liechtenstein
- Lithuania
- Luxembourg
- Macau
- Macedonia

- Madagascar
- Malawi
- Malaysia
- Maldives
- Mali
- Malta
- Marshall Islands
- Martinique
- Mauritania
- Mauritius
- Mayotte
- Mexico
- Micronesia, Fed. St.
- Moldova
- Monaco
- Mongolia
- Montserrat
- Morocco
- Mozambique
- Namibia
- Nauru
- Nepal
- Netherlands
- Netherlands Antilles
- New Caledonia
- New Zealand
- Nicaragua
- Niger
- Nigeria
- N. Mariana Islands
- Norway
- Oman
- Pakistan
- Palau
- Panama
- Papua New Guinea
- Paraguay
- Peru
- Philippines
- Poland
- Portugal
- Puerto Rico

- Qatar
- Reunion
- Romania
- Russia
- Rwanda
- Saint Helena
- Saint Kitts & Nevis
- Saint Lucia
- St Pierre & Miquelon
- Saint Vincent and the Grenadines
- Samoa
- San Marino
- Sao Tome & Principe
- Saudi Arabia
- Senegal
- Serbia
- Seychelles
- Sierra Leone
- Singapore
- Slovakia
- Slovenia
- Solomon Islands
- Somalia
- South Africa
- Spain
- Sri Lanka
- Sudan
- Suriname
- Swaziland
- Sweden
- Switzerland
- Syria
- Taiwan
- Tajikistan
- Tanzania
- Thailand
- Togo
- Tonga
- Trinidad & Tobago
- Tunisia
- Turkey
- Turkmenistan

- Turks & Caicos Is
- Tuvalu
- Uganda
- Ukraine
- United Arab Emirates
- United Kingdom
- United States
- Uruguay
- Uzbekistan
- Vanuatu
- Venezuela
- Vietnam
- Virgin Islands
- Wallis and Futuna
- West Bank
- Western Sahara
- Yemen
- Zambia
- Zimbabwe

2. **2. What type of institution do you work for?** *

Check all that apply.

- [ ] National Malaria Control Program
- [ ] Ministry of health
- [ ] Vector-Borne Disease Control Program
- [ ] National Center for Disease Control and Public Health
- [ ] Global Fund Malaria Program
- [ ] Local or Regional WHO Office (OMS)
- [ ] World Health Organization (WHO / OMS)
- [ ] University/ Educational Institution
- [ ] Other: ____________________

MALARIA TESTING: PROGRAM GOALS AND CHALLENGES

3. **3. Malaria diagnosis is important to case management, surveillance, and elimination activities. Today, which of the following best reflects your national malaria program's goals for malaria testing? Please select one** *

Mark only one oval.

- 3.1 The program is primarily focused on increasing testing rates, diagnosing as many febrile people with suspected malaria as possible, and treating them appropriately (i.e. expanding access to high quality case management) *Skip to question 4.*
- 3.2 The program is focused primarily on testing related to elimination or prevention of reintroduction, for example: surveillance, shrinking the reservoir by identifying foci of infection and responding to them; avoiding outbreaks *Skip to question 11.*
- 3.3 The program is focused on both of the above, it depends on the region of the country *Skip to question 12.*

MALARIA TESTING: PROGRAM GOALS AND CHALLENGES

4. **3.1.1. Approximately what percentage of patients in your country seek care from a community health worker? (If you do not have data, please provide your best estimate.)** *

Mark only one oval.

- 0-5% of patients
- 6-10% of patients
- 11-15% of patients
- 16-20% of patients
- >21% of patients

5. **3.1.2. In some countries, the private sector, both formal and retail, performs malaria testing. Please provide your best guess of the number of tests (RDTs and microscopy) the private sector in your country performs each year.** *

Mark only one oval per row.

	Does not perform any testing	1-100,000 tests	101,000-999,999 tests	1 million-4.9 million tests	5 million – 20 million tests	>20 million tests
Formal private sector (e.g., private hospitals, clinics and laboratories)						
Retail private sector (e.g., pharmacies, drug shops, etc.)						

6. **3.1.3. What could get in the way of the program reaching its goals around testing? Please select up to three items from the list below that you consider to be the most important.** *

Check all that apply.

- [ ] Problems with microscopy (any type of problem: training staff, quality, supply chain, etc.)
- [ ] Problem related to rapid test products (e.g., ability to pick up asymptomatic infections, HRP2 deletions, speciation, persistence of antigens etc.)
- [ ] Challenges maintaining the quality of malaria RDT testing (e.g.,challenges with training related to switching RDTs, staff turnover; test operators not following SOPs or misinterpreting results)
- [ ] RDT stock outs and supply chain issues
- [ ] Coverage: not enough points of service near patients (e.g.,not enough CHW or health posts in rural areas that perform RDTs etc.)
- [ ] Health worker case management related challenges (e.g., health workers do not follow guidelines – fail to test all suspected cases, do not accept malaria test results, do not know how to manage negative RDTs)
- [ ] Patient acceptance of testing and test results is too low
- [ ] Many patients seek care from providers that do not offer testing (e.g.,informal private sector, alternative/traditional providers)
- [ ] Challenges related to funding testing services (supplies, RDTs, training, quality, etc)
- [ ] Challenges related to blood based testing (e.g., safe handling of blood and sharps, waste disposal)
- [ ] Problems related to reporting of testing results, compiling surveillance data
- [ ] Other: ____________________

7. **3.1.4. Please consider the perspective of health workers in the public and private sector (e.g., doctors, nurses, community health workers, pharmacists). To what extent do any of the following limit achieving the national program's goals for malaria testing and treatment? Please Indicate one response for each row.** *

Mark only one oval per row.

	Not limiting at all	Somewhat limiting	Very limiting
Perception that testing takes too much time, given workload	○	○	○
There are not enough qualified staff to perform RDTs or microscopy	○	○	○
Reluctance to manage patients based on test results (e.g.,patient expectations for treatment, not wanting to miss dangerous infection; trust clinical judgement/experience over test results)	○	○	○
Inconsistent supply of RDTs prevents clinicians from testing	○	○	○
Pharmacists / drug shop workers are reluctant to perform blood based testing	○	○	○
Health workers do not trust test accuracy (microscopy or RDTs)	○	○	○
Health worker attitude: not concerned with quality of testing or case management	○	○	○
Difficulty collecting samples from the patients	○	○	○

8. **Other (please specify)**

____

Skip to question 19.

MALARIA TESTING: PROGRAM GOALS AND CHALLENGES

9. **3.1.5. Please consider the perspective of patients, to what extent do any of the following limit achieving the national programs target's and goals for malaria testing and treatment? Please Indicate one response for each row.** *
Mark only one oval per row.

	Not limiting at all	Somewhat limiting	Very limiting
Cultural, religious, traditional reasons for avoiding having blood drawn	⬭	⬭	⬭
Children prefer to avoid fingerpricks	⬭	⬭	⬭
Adults prefer to avoid fingerpricks	⬭	⬭	⬭
People avoid testing because of long waiting times for the results	⬭	⬭	⬭
Patients expect an antimalarial treatment, regardless of test results	⬭	⬭	⬭
There are not enough points of service with testing near to populations who need testing	⬭	⬭	⬭
Convenience of testing: clinicians with testing are not available during convenient hours and/or convenient to home /transport.	⬭	⬭	⬭
Cost of testing is perceived to be high, both direct and indirect costs (e.g. lost time at work)	⬭	⬭	⬭
Patients think that blood is taken for HIV testing, not for malaria testing	⬭	⬭	⬭
Patient preference to self-treat/ self-medicate	⬭	⬭	⬭
Due to religious, cultural, or traditional beliefs, patients do not seek care from clinicians who offer testing	⬭	⬭	⬭
Patients do not trust the accuracy and quality of microscopy or RDTs.	⬭	⬭	⬭
Stock outs of tests and treatments are common among clinicians that can perform testing; patients therefore tend to avoid these facilities	⬭	⬭	⬭

10. **Other (please specify)**

____

Skip to question 19.

MALARIA TESTING: PROGRAM GOALS AND CHALLENGES

11. **3.2.1. What could get in the way of the program reaching its goals around testing? Please select up to three items from the list below that you consider to be the most important.** *

Check all that apply.

- [ ] Problems with microscopy (any type of problem: training staff, quality, supply chain, etc.)
- [ ] Problem related to rapid test products (e.g., ability to pick up asymptomatic infections, HRP2 deletions, speciation, etc.)
- [ ] Challenges maintaining the quality of malaria RDT testing (e.g., challenges with training related to switching RDTs, staff turnover; test operators not following SOPs or misinterpreting results)
- [ ] RDT stock outs and supply chain issues
- [ ] Coverage: not enough points of service near patients (e.g.,not enough CHW or health posts in rural areas that perform RDTs etc.)
- [ ] Health worker case management related challenges (e.g., health workers do not follow guidelines – fail to test all suspected cases, do not accept malaria test results, do not know how to manage negative RDTs)
- [ ] Patient acceptance of testing and test results is low
- [ ] Patients who have no symptoms are reluctant to be tested – for example during reactive case detection or surveys.
- [ ] Many patients seek care from providers that do not offer testing (e.g., informal private sector, alternative/traditional providers)
- [ ] Challenges related to funding for active case detection and surveillance activities
- [ ] Challenges related to funding testing (supplies, training, quality, etc)
- [ ] Challenges related to blood based testing (e.g., safe handling of blood and sharps, waste disposal)
- [ ] Problems related to reporting of testing results, compiling surveillance data
- [ ] Other: ____________________

Skip to question 19.

MALARIA TESTING: PROGRAM GOALS AND CHALLENGES

12. **3.3.1. Approximately what percentage of patients in your country seek care from a community health worker? (If you do not have data, please provide your best estimate)** *

Mark only one oval.

- ( ) 0-5% of patients
- ( ) 6-10% of patients
- ( ) 11-15% of patients
- ( ) 16-20% of patients
- ( ) >21% of patients

13. **3.3.2. In some countries, the private sector, both formal and retail, performs malaria testing. Please provide your best guess of the number of tests (RDTs and microscopy) the private sector in your country performs each year.** *

Mark only one oval per row.

	Does not perform any testing	1-100,000 tests	101,000-999,999 tests	1 million-4.9 million tests	5 million – 20 million tests	>20 million tests
Formal private sector (e.g., private hospitals, clinics and laboratories)	◯	◯	◯	◯	◯	◯
Retail private sector (e.g., pharmacies, drug shops, etc.)	◯	◯	◯	◯	◯	◯

14. **3.3.3. What could get in the way of the program reaching its goals around testing? Please select up to three items from the list below that you consider to be the most important.** *

Check all that apply.

- [ ] Problems with microscopy (any type of problem: training staff, quality, supply chain, etc.)
- [ ] Problem related to rapid test products (e.g., ability to pick up asymptomatic infections, HRP2 deletions, speciation, persistence of antigens etc.)
- [ ] Challenges maintaining the quality of malaria RDT testing (e.g.,challenges with training related to switching RDTs, staff turnover; test operators not following SOPs or misinterpreting results)
- [ ] RDT stock outs and supply chain issues
- [ ] Coverage: not enough points of service near patients (e.g.,not enough CHW or health posts in rural areas that perform RDTs etc.)
- [ ] Health worker case management related challenges (e.g., health workers do not follow guidelines – fail to test all suspected cases, do not accept malaria test results, do not know how to manage negative RDTs)
- [ ] Patient acceptance of testing and test results is too low
- [ ] Many patients seek care from providers that do not offer testing (e.g.,informal private sector, alternative/traditional providers)
- [ ] Challenges related to funding testing services (supplies, RDTs, training, quality, etc)
- [ ] Challenges related to blood based testing (e.g., safe handling of blood and sharps, waste disposal)
- [ ] Problems related to reporting of testing results, compiling surveillance data
- [ ] Other: ______________________

15. **3.3.4. Please consider the perspective of health workers in the public and private sector (e.g., doctors, nurses, community health workers, pharmacists). To what extent do any of the following limit achieving the national program's goals for malaria testing and treatment? Please Indicate one response for each row.** *

Mark only one oval per row.

	Not limiting at all	Somewhat limiting	Very limiting
Perception that testing takes too much time, given workload			
There are not enough qualified staff to perform RDTs or microscopy			
Reluctance to manage patients based on test results (e.g.,patient expectations for treatment, not wanting to miss dangerous infection; trust clinical judgement/experience over test results)			
Inconsistent supply of RDTs prevents clinicians from testing			
Pharmacists / drug shop workers are reluctant to perform blood based testing			
Health workers do not trust test accuracy (microscopy or RDTs)			
Health worker attitude: not concerned with quality of testing or case management			
Difficulty collecting samples from the patients			

16. **Other (please specify)**

17. **3.3.5. Please consider the perspective of patients, to what extent do any of the following limit achieving the national programs target's and goals for malaria testing and treatment? Please Indicate one response for each row.** *

Mark only one oval per row.

	Not limiting at all	Somewhat limiting	Very limiting
Cultural, religious, traditional reasons for avoiding having blood drawn			
Children prefer to avoid fingerpricks			
Adults prefer to avoid fingerpricks			
People avoid testing because of long waiting times for the results			
Patients expect an antimalarial treatment, regardless of test results			
There are not enough points of service with testing near to populations who need testing			
Convenience of testing: clinicians with testing are not available during convenient hours and/or convenient to home /transport.			
Cost of testing is perceived to be high, both direct and indirect costs (e.g. lost time at work)			
Patients think that blood is taken for HIV testing, not for malaria testing			
Patient preference to self-treat/ self-medicate			
Due to religious, cultural, or traditional beliefs, patients do not seek care from clinicians who offer testing			
Patients do not trust the accuracy and quality of microscopy or RDTs.			
Stock outs of tests and treatments are common among clinicians that can perform testing; patients therefore tend to avoid these facilities			

18. **Other (please specify)**

MALARIA TESTING: PROGRAM GOALS AND CHALLENGES

19. **In thinking about your national program's goals and priorities with respect to testing, are there any specific targets for malaria diagnosis, or areas of particular focus, either current or in the near future? Please describe:**

POTENTIAL USE SCENARIOS / KEY POPULATIONS FOR NON-INVASIVE TESTS

This section asks for your feedback on potential uses of non-invasive tests and on user preferences for blood, urine and saliva sampling.

20. **4. In your country, in which of the following situations could non-invasive tests that use either saliva or urine samples be useful?** *

Mark only one oval per row.

	Definitely not useful	Probably not useful	Probably useful	Definitely useful	Not applicable – country would not do this type of testing
Malaria testing in heath facilities	◯	◯	◯	◯	◯
Malaria testing by community health workers	◯	◯	◯	◯	◯
Malaria testing in the retail private sector (pharmacies, drug shops)	◯	◯	◯	◯	◯
Reactive case detection	◯	◯	◯	◯	◯
Border screening	◯	◯	◯	◯	◯
Proactive screening of high risk populations or locations	◯	◯	◯	◯	◯
Malaria testing during surveys and surveillance	◯	◯	◯	◯	◯
Self-testing (e.g.,home kits)	◯	◯	◯	◯	◯
Malaria testing during high risk disease outbreaks (e.g.,Ebola)	◯	◯	◯	◯	◯
Screening pregnant women	◯	◯	◯	◯	◯
Malaira testing in children	◯	◯	◯	◯	◯

21. **Other (please specify)**

____

22. **4.1. If you selected self-testing as probably or definitely useful, please describe which population groups and settings would benefit from self-testing?**

____
____
____
____
____

23. **5. Please use your best judgment to predict how the following populations would react to a SALIVA based malaria test if it existed today** *

Mark only one oval per row.

	Readily accept saliva test	May hesitate to switch to saliva test at first, but eventually accept	Prefer blood based test, unlikely to use saliva test	Not applicable to our country
Children				
Adults				
Migrant/mobile populations				
Indigenous people				
Remote/rural communities				
Populations being tested as part of a survey (mostly asymptomatic) for surveillance purposes				
Professional health care workers (e.g. doctors, nurses)				
Community health workers				
Retailers (drug shops, pharmacists)				

24. **6. Please use your best judgment to predict how the following populations would react to a URINE based malaria test if it existed today** *

Mark only one oval per row.

	Readily accept urine test	May hesitate to switch to urine test at first, but eventually accept	Prefer blood based test, unlikely to use urine test	Not applicable to our country
Children	◯	◯	◯	◯
Adults	◯	◯	◯	◯
Pregnant women	◯	◯	◯	◯
Migrant/mobile populations	◯	◯	◯	◯
Indigenous people	◯	◯	◯	◯
Remote/rural communities	◯	◯	◯	◯
Populations being tested as part of a survey (mostly asymptomatic) for surveillance purposes	◯	◯	◯	◯
Professional health care workers (e.g. doctors, nurses)	◯	◯	◯	◯
Community health workers	◯	◯	◯	◯
Retailers (drug shops, pharmacists)	◯	◯	◯	◯

25. **7. Please share any insights related to cultural beliefs about blood draws, or patient expectations about blood testing and malaria in your country and how these could affect patient preferences for blood based tests or non-invasive malaria tests**

____

____

____

____

____

POTENTIAL USE SCENARIOS / KEY POPULATIONS FOR NON-INVASIVE TESTS

26. **8. Assuming they perform equally well, which sample for a malaria test would be your program's first choice?** *

Mark only one oval.

- ◯ Urine
- ◯ Saliva
- ◯ Blood

27. **9. Assuming they perform equally well, which sample for a malaria test would be your program's second choice?** *

Mark only one oval.

- Urine
- Saliva
- Blood

28. **10. Please consider the public health impact of non-invasive tests in your country, and indicate your level of agreement with the following statements:** *

Mark only one oval per row.

	Strongly disagree	Disagree	Agree	Strongly agree	Not applicable to our country
Malaria testing is already minimally invasive, well-accepted, and common in our country, there is no need to change to non-invasive tests unless new tests have other advantages (e.g., significantly improved performance, turn-around time etc.)					
Non-invasive tests could significantly increase access to testing in those populations that we struggle to reach.					
Non-invasive tests could significantly increase access to testing in our country overall.					
Expanding the number of people who can conduct testing is important to our program.					
Non-invasive tests could increase testing in the retail private sector.					
Self-testing (e.g. testing at home) would allow patients to seek care for malaria earlier.					
There are no problems with the use of blood for malaria testing in our country					

SALIVA TEST: ADOPTION AND PRICE

For this section, please assume that a SALIVA malaria test is:
- As sensitive as traditional malaria RDT, however it does detect parasites with HRP2 deletions and it correlates well with active infection.
- As portable and easy to use as a malaria RDT (e.g.,disposable, requires minimal operator training or processing steps, results available in <30 minutes).
- Can have either one or two test lines (species as appropriate for your country).
- Provides a qualitative result.

29. **11. Please assume that price is not an issue and that this type of saliva test is available today. How likely is your program to implement this test?** *
Mark only one oval.

- Extremely likely *Skip to question 30.*
- Very likely *Skip to question 30.*
- Not so likely *Skip to question 31.*
- Not at all likely *Skip to question 31.*

SALIVA TEST: ADOPTION AND PRICE

30. **11.1. Are there any new activities that your program would consider doing if a saliva test were available? Please select those activities from the list below that the program might start to do if a saliva test were available:** *
Check all that apply.

- Malaria testing by community health workers
- Malaria testing in the retail private sector (e.g., drug shops and pharmacies)
- Reactive case detection
- Border screening
- Proactive screening
- Additional surveys/surveillance activities that involve testing
- Self-testing (e.g., home test kits available to the population)
- Limited Self-testing (e.g., provided under the public sector for hard to reach populations, people living in remote areas, or to the population during high risk outbreaks)
- Travelers self-testing kits
- Malaria testing by health workers during high risk disease outbreaks (e.g., Ebola)
- Other: ____________________

Skip to question 32.

SALIVA TEST: ADOPTION AND PRICE

31. **11.2. What is the main reason that the program is not likely to implement a non-invasive saliva test? Please select the response that best fits** *
Mark only one oval.

- We are generally satisfied with current diagnostic tests
- General lack of confidence in saliva based testing
- Clinicians are unlikely to accept this test compared to blood tests
- Patients are unlikely to accept this test compared to blood tests
- For any new malaria tests, we prefer more than 2 test lines for species differentiation
- For any new malaria tests, we prefer quantitative results
- For any new malaria tests, we prefer test that can detect asymptomatic infections
- We expect the saliva test to cost more than current tests, and as a result it will not be affordable to the program
- Other: ____________________

SALIVA TEST: ADOPTION AND PRICE

32. **12. Please indicate how your program might use the saliva test at the following prices. This question is meant to help us understand adoption and use at different prices, we recognize that it is difficult to answer, so please provide your best estimate. Please, make one selection for each row** *

Mark only one oval per row.

	Probably not use	Very limited use (e.g., selected situations, populations)	Would partially replace some of the testing that we currently perform	Would largely replace the RDTs we use today	Would largely replace both the RDTs and microscopy we use today
US$0.0-0.50	◯	◯	◯	◯	◯
US$0.51-1.00	◯	◯	◯	◯	◯
US$1.01-1.50	◯	◯	◯	◯	◯
US>$1.51	◯	◯	◯	◯	◯

33. **13. Now assume that this saliva test is more sensitive than a traditional RDT and is able to detect sub-microscopic infections?** *

Mark only one oval per row.

	Probably not use	Very limited use (e.g., selected situations, populations)	Would partially replace some of the testing that we currently perform	Would largely replace the RDTs we use today	Would largely replace both the RDTs and microscopy we use today
US$0.51-1.00	◯	◯	◯	◯	◯
US$1.01-1.50	◯	◯	◯	◯	◯
US>$1.51	◯	◯	◯	◯	◯

URINE TEST: ADOPTION AND PRICE

For this section, please assume that an URINE malaria test is:
- As sensitive as traditional malaria RDT, however it does detect parasites with HRP2 deletions and it correlates well with active infection.
- As portable and easy to use as a malaria RDT (e.g.,disposable, requires minimal operator training or processing steps, results available in <30 minutes).
- Can have either one or two test lines (species as appropriate for your country).
- Provides a qualitative result.

34. **14. Please assume that price is not an issue and that this type of unine test is available today. How likely is your program to implement this test?** *

Mark only one oval.

- ◯ Extremely likely *Skip to question 35.*
- ◯ Very likely *Skip to question 35.*
- ◯ Not so likely *Skip to question 36.*
- ◯ Not at all likely *Skip to question 36.*

URINE TEST: ADOPTION AND PRICE

35. **14.1. Are there any new activities that your program would consider doing if a urine test were available? Please select those activities from the list below that the program might start to do if a urine test were available:** *

Check all that apply.

- [ ] Malaria testing by community health workers
- [ ] Malaria testing in the retail private sector (e.g.,drug shops and pharmacies)
- [ ] Reactive case detection
- [ ] Border screening
- [ ] Proactive screening
- [ ] Additional surveys/surveillance activities that involve testing
- [ ] Self-testing (e.g.,home test kits available to the population)
- [ ] Limited Self-testing (e.g.,provided under the public sector for hard to reach populations, people living in remote areas, or to the population during high risk outbreaks)
- [ ] Travelers self-testing kits
- [ ] Malaria testing by health workers during high risk disease outbreaks (e.g.,Ebola)
- [ ] Other: ____________________

Skip to question 37.

URINE TEST: ADOPTION AND PRICE

36. **14.2. What is the main reason that the program is not likely to implement the non-invasive urine test? Please select the response that best fits.** *

Mark only one oval.

- ( ) We are generally satisfied with current diagnostic tests
- ( ) General lack of confidence in urine based testing
- ( ) Clinicians are less likely to accept this test compared to blood tests
- ( ) Patients are unlikely to accept this test compared to blood tests
- ( ) For any new malaria tests, we prefer more than 2 test lines for species differentiation
- ( ) For any new malaria tests, we prefer quantitative results
- ( ) For any new malaria tests, we prefer test that can detect asymptomatic infections
- ( ) We expect the urine test to cost more than current tests, and as a result it will not be affordable to the program
- ( ) Other: ____________________

URINE TEST: ADOPTION AND PRICE

37. **15. Please indicate how your program might use a urine test at the following prices. This question is meant to help us understand adoption and use at different prices, we recognize that it is difficult to answer, so please provide your best estimate. Please, make one selection for each row** *

Mark only one oval per row.

	Probably not use	Very limited use (e.g., selected situations, populations)	Would partially replace some of the testing that we currently perform	Would largely replace the RDTs we use today	Would largely replace both the RDTs and microscopy we use today
US$0.0-0.50	◯	◯	◯	◯	◯
US$0.51-1.00	◯	◯	◯	◯	◯
US$1.01-1.50	◯	◯	◯	◯	◯
US>$1.51	◯	◯	◯	◯	◯

38. **16. Now assume that this urine test is more sensitive than a traditional RDT and is able to detect sub-microscopic infections** *

Mark only one oval per row.

	Probably not use	Very limited use (e.g., selected situations, populations)	Would partially replace some of the testing that we currently perform	Would largely replace the RDTs we use today	Would largely replace both the RDTs and microscopy we use today
US$0.51-1.00	◯	◯	◯	◯	◯
US$1.01-1.50	◯	◯	◯	◯	◯
US>$1.51	◯	◯	◯	◯	◯

PREFERENCES FOR NON-INVASIVE TESTS

39. **17. A variety of test types are possible (i.e. species detection combinations). Assume that two test lines are possible for a non-invasive test. What test configuration would be most useful for your program? *Assume Pf detects all P. falciparum parasites, even HRP2 deleted parasites** *

Mark only one oval.

- ◯ Pan/Pf
- ◯ Pf/Pv
- ◯ Pan/Pv
- ◯ Pan/Pvo
- ◯ Pf/Pvo
- ◯ Other: ______________________

40. **18. Please indicate your program's preferences for the following in a new malaria test:** *

Mark only one oval per row.

	Absolutely essential	Nice to have; optimal	Probably not needed
Stable at 40°C	◯	◯	◯
18 month shelf life	◯	◯	◯
24 month shelf life	◯	◯	◯
Faster time to results than today's RDTs	◯	◯	◯
Able to detect submicroscopic infections	◯	◯	◯
Quantitative result	◯	◯	◯
Could be used by untrained lay people (following simple pictorial instructions)	◯	◯	◯

41. **19. This question is about electronic test readers: A reader is a small, hand held device, that is battery operated and holds charge for approximately 24 hrs. Readers can perform multiple functions, including: i) enhance sensitivity of the test; ii) automate data capture (test usage, results, GPS coordinates) and transfer data to a central database with analytical software; and iii) improve quality by guiding test processing and interpreting results. Drawbacks include increased cost and possibly decreased access due to the addition of a device. Based on this, how likely would your program be to implement a non-invasive test with a reader?** *

Mark only one oval.

- ◯ Extremely likely
- ◯ Very likely
- ◯ Not so likely
- ◯ Not at all likely

END SURVEY

Thank you for participating in this survey, we really appreciate your time and thoughtful consideration of our questions. Your perspective is invaluable to this initiative and will inform decisions about the next steps in the development of new malaria diagnostics as well as in how donors and groups like FIND might support malaria testing more generally - for case management, surveillance, and elimination.

42. **If you have any additional thoughts, please provide them in the comment box below.**

43. **We are also developing a separate, brief survey for health care workers in order to gather their perspective on blood, saliva and urine tests. If you could provide the names and email addresses of a few health care workers in your country who might be willing to perform the survey that would be extremely helpful. We would welcome input from the nursing staff, IMCI, CHW programs, clinicians at local private sector facilities.**

Powered by
Google Forms
