# Supplementary material for: Acceptance and perceived value of non-invasive malaria diagnostic tests in malaria-endemic countries
Source: Malar J. 2021 Sep 24;20:379. doi: 10.1186/s12936-021-03911-y (PMC8461884; doi:10.1186/s12936-021-03911-y)
Supplement: Supplementary file 2 — Additional file 2. Structured questionnaire in French. [file 12936_2021_3911_MOESM2_ESM.pdf]

ENQUÊTE SUR LES TESTS DIAGNOSTIQUES NON INVASIFS POUR LE PALUDISME

* Required

FIND
Because diagnosis matters

Nous vous remercions de participer à cette enquête

INTRODUCTION

La Fondation Bill et Melinda Gates envisage de soutenir le développement de tests diagnostiques non invasifs pour le paludisme. Ces tests seraient similaires aux TDR actuels, sauf qu'ils se feraient à partir d'un échantillon de salive ou d'urine et non de sang. Avant d'investir des sommes importantes dans le développement de tels tests, la Fondation nous a demandé de les aider à mieux comprendre le marché des tests non invasifs, et d'estimer tout impact potentiel que ceux-ci pourraient avoir sur la lutte contre le paludisme et les objectifs d'élimination à l'échelle mondiale.

C'est dans ce contexte que nous aimerions avoir votre avis sur plusieurs questions regroupées en quatre sections :

1. Les priorités du programme de lutte contre le paludisme et les défis liés aux tests
2. Les scénarios d'utilisations potentielles et les populations clés visées par les tests non invasifs
3. Les avis sur l'adoption et les prix
4. Les préférences concernant plusieurs caractéristiques clés des produits

Pour répondre à ces questions, veuillez tenir compte de la situation dans votre pays ainsi que de l'expérience de votre programme national. Vos réponses seront utilisées sous forme agrégée, et aucune évaluation des réponses individuelles ne sera faite. Soyez le plus honnête possible – il n'y a pas de bonne ou de mauvaise réponse.
Nous savons qu'il n'est pas facile de se prononcer sur un produit qui n'existe pas encore et que vous pourriez ne pas connaître la réponse à certaines questions de l'enquête. Nous voulons recueillir vos hypothèses et vos prédictions éclairées, basées sur votre expérience.

L'enquête devrait prendre environ 20 minutes. Nous vous remercions par avance pour votre précieuse collaboration.

DONNÉES DÉMOGRAPHIQUES

Cette enquête est anonyme, mais nous aimerions pouvoir analyser les données par région et classer les réponses par type d'établissement (par ex. réponses des programmes nationaux de lutte contre le paludisme).

1. **1. Dans quel pays travaillez-vous actuellement ? (Si vous travaillez dans plusieurs pays différents, veuillez choisir celui où vous passez le plus de temps).** *

Mark only one oval.

- Afghanistan
- Albania
- Algeria
- American Samoa
- Andorra
- Angola
- Anguilla
- Antigua & Barbuda
- Argentina
- Armenia
- Aruba
- Australia
- Austria
- Azerbaijan
- Bahamas, The
- Bahrain
- Bangladesh
- Barbados
- Belarus
- Belgium
- Belize
- Benin
- Bermuda
- Bhutan
- Bolivia
- Bosnia & Herzegovina
- Botswana
- Brazil
- British Virgin Is.
- Brunei
- Bulgaria
- Burkina Faso
- Burma
- Burundi
- Cambodia
- Cameroon
- Canada
- Cape Verde
- Cayman Islands
- Central African Rep.

- Chad
- Chile
- China
- Colombia
- Comoros
- Congo, Dem. Rep.
- Congo, Repub. of the
- Cook Islands
- Costa Rica
- Cote d'Ivoire
- Croatia
- Cuba
- Cyprus
- Czech Republic
- Denmark
- Djibouti
- Dominica
- Dominican Republic
- East Timor
- Ecuador
- Egypt
- El Salvador
- Equatorial Guinea
- Eritrea
- Estonia
- Ethiopia
- Faroe Islands
- Fiji
- Finland
- France
- French Guiana
- French Polynesia
- Gabon
- Gambia, The
- Gaza Strip
- Georgia
- Germany
- Ghana
- Gibraltar
- Greece
- Greenland
- Grenada

- Guadeloupe
- Guam
- Guatemala
- Guernsey
- Guinea
- Guinea-Bissau
- Guyana
- Haiti
- Honduras
- Hong Kong
- Hungary
- Iceland
- India
- Indonesia
- Iran
- Iraq
- Ireland
- Isle of Man
- Israel
- Italy
- Jamaica
- Japan
- Jersey
- Jordan
- Kazakhstan
- Kenya
- Kiribati
- Korea, North
- Korea, South
- Kuwait
- Kyrgyzstan
- Laos
- Latvia
- Lebanon
- Lesotho
- Liberia
- Libya
- Liechtenstein
- Lithuania
- Luxembourg
- Macau
- Macedonia

- Madagascar
- Malawi
- Malaysia
- Maldives
- Mali
- Malta
- Marshall Islands
- Martinique
- Mauritania
- Mauritius
- Mayotte
- Mexico
- Micronesia, Fed. St.
- Moldova
- Monaco
- Mongolia
- Montserrat
- Morocco
- Mozambique
- Namibia
- Nauru
- Nepal
- Netherlands
- Netherlands Antilles
- New Caledonia
- New Zealand
- Nicaragua
- Niger
- Nigeria
- N. Mariana Islands
- Norway
- Oman
- Pakistan
- Palau
- Panama
- Papua New Guinea
- Paraguay
- Peru
- Philippines
- Poland
- Portugal
- Puerto Rico

- Qatar
- Reunion
- Romania
- Russia
- Rwanda
- Saint Helena
- Saint Kitts & Nevis
- Saint Lucia
- St Pierre & Miquelon
- Saint Vincent and the Grenadines
- Samoa
- San Marino
- Sao Tome & Principe
- Saudi Arabia
- Senegal
- Serbia
- Seychelles
- Sierra Leone
- Singapore
- Slovakia
- Slovenia
- Solomon Islands
- Somalia
- South Africa
- Spain
- Sri Lanka
- Sudan
- Suriname
- Swaziland
- Sweden
- Switzerland
- Syria
- Taiwan
- Tajikistan
- Tanzania
- Thailand
- Togo
- Tonga
- Trinidad & Tobago
- Tunisia
- Turkey
- Turkmenistan

- Turks & Caicos Is
- Tuvalu
- Uganda
- Ukraine
- United Arab Emirates
- United Kingdom
- United States
- Uruguay
- Uzbekistan
- Vanuatu
- Venezuela
- Vietnam
- Virgin Islands
- Wallis and Futuna
- West Bank
- Western Sahara
- Yemen
- Zambia
- Zimbabwe

2. **2. Pour quel type d'établissement travaillez-vous ?** *

Check all that apply.

- [ ] Programme National de Lutte contre le Paludisme
- [ ] Ministère de la santé
- [ ] Programme de Lutte contre les Maladies à Transmission Vectorielle
- [ ] Centre National de Lutte contre les Maladies et de Santé Publique
- [ ] Programme de Lutte contre le Paludisme du Fond Mondial
- [ ] Bureau Local ou Régional de l'OMS
- [ ] Organisation mondiale de la Santé (OMS)
- [ ] Université/ Établissement d'Enseignement
- [ ] Other: ____________________

TESTS DIAGNOSTIQUES POUR LE PALUDISME : OBJECTIFS ET DÉFIS DU PROGRAMME

3. **3. Il est important de pouvoir diagnostiquer le paludisme pour assurer la prise en charge et la surveillance des cas, ainsi que les activités d'élimination. Parmi les énoncés ci-dessous, lequel correspond le mieux, selon vous, aux objectifs actuels de votre programme national de lutte contre le paludisme en termes de diagnostic ?** *

Mark only one oval.

- 3.1 Le programme vise principalement à augmenter la fréquence des tests, à dépister le plus de personnes fébriles suspectées de paludisme possible, et à les traiter de manière appropriée (c'est-à-dire élargir l'accès à une prise en charge de qualité). *Skip to question 4.*
- 3.2 Le programme s'occupe principalement des tests liés à l'élimination ou à la prévention de la réintroduction, par ex. via des activités de surveillance, de réduction du réservoir en identifiant les foyers d'infection et en prenant les mesures appropriées, ou de prévention des flambées. *Skip to question 11.*
- 3.3 Le programme intègre les deux approches ci-dessus, en fonction des régions dans chaque pays. *Skip to question 12.*

TESTS DIAGNOSTIQUES POUR LE PALUDISME : OBJECTIFS ET DÉFIS DU PROGRAMME

4. **3.1.1. Environ quel pourcentage de patients dans votre pays consultent des agents de santé communautaire ? (Si vous ne connaissez pas la réponse, donner votre meilleure estimation)** *

Mark only one oval.

- 0-5% des patients
- 6-10% des patients
- 11-15% des patients
- 16-20% des patients
- >21% des patients

5. **3.1.2. Dans certains pays, les tests diagnostiques pour le paludisme sont effectués par les secteurs privés formel et de détail. Estimez au mieux le nombre de tests (TDR et microscopie) effectués chaque année par le secteur privé dans votre pays.** *

Mark only one oval per row.

	Aucun test effectué	1-100.000 tests	101.000-999.999 tests	1 million- 4,9 millions de tests	5 millions – 20 millions de tests	>20 millions de tests
Secteur privé formel (par ex. hôpitaux, cliniques et laboratoires privés)						
Secteur privé de détail (par ex. pharmacies, parapharmacies, etc.)						

6. **3.1.3. Quels obstacles pourraient empêcher la réalisation des objectifs du programme en termes de diagnostic ? Veuillez sélectionner les propositions les plus importantes, selon vous, dans la liste ci-dessous (maximum trois).** *

Check all that apply.

- [ ] Tout type de problème lié à la microscopie (formation du personnel, qualité, chaîne d'approvisionnement, etc.)
- [ ] Problème lié aux produits de diagnostic rapide (par ex. capacité à détecter des infections asymptomatiques, délétion du gène codant pour la protéine HRP2, détermination de l'espèce, persistance d'antigènes, etc.)
- [ ] Défis liés au maintien de la qualité des TDR pour le paludisme (par ex. défis liés à la formation sur le changement de TDR, les roulements de personnel, tests effectués sans respecter les POS ou mauvaise interprétation des résultats)
- [ ] Ruptures de stock de TDR et problèmes liés à la chaîne d'approvisionnement
- [ ] Couverture : nombre insuffisant de points de service à proximité des patients (par ex. pas assez d'agents de santé communautaire ou de postes de santé dans les zones rurales effectuant des TDR, etc.)
- [ ] Défis liés à la prise en charge par les agents de santé (par ex. directives non suivies – tous les cas suspects ne sont pas testés, les résultats du test de diagnostic du paludisme ne sont pas acceptés, ils ne savent pas comment gérer les TDR négatifs)
- [ ] Le degré d'acceptation des tests et des résultats des tests est trop faible
- [ ] De nombreux patients s'adressent à des prestataires de soins qui ne proposent pas de tests diagnostiques (par ex. secteur privé informel, prestataire alternatif/traditionnel)
- [ ] Défis liés au financement du diagnostic (fournitures, TDR, formation, qualité, etc.)
- [ ] Défis liés aux tests sur échantillon de sang (par ex. sécurité lors de la manipulation du sang et des objets tranchants, élimination des déchets)
- [ ] Problèmes liés à la notification des résultats des tests, compilation des données de surveillance
- [ ] Other: ______________________

7. **3.1.4. En vous plaçant du point de vue des agents de santé des secteurs public et privé (par ex. médecins, infirmiers, agents de santé communautaire, pharmaciens), dans quelle mesure les facteurs ci-dessous gênent-ils la réalisation des objectifs du programme national en termes de diagnostic et de traitement du paludisme ? Veuillez sélectionner une seule réponse pour chaque rangée.** *

Mark only one oval per row.

	Pas du tout	Un peu	Beaucoup
Sentiment que le test prend trop temps étant donné la charge de travail	⬭	⬭	⬭
Il n'y a pas assez de personnes qualifiées pour effectuer les TDR ou la microscopie	⬭	⬭	⬭
Réticence à appliquer les résultats des tests dans la prise en charge (par ex. patient exigeant un traitement, peur de passer à côté d'une infection dangereuse, confiance plus importante dans l'appréciation clinique /expérience que dans les résultats du test)	⬭	⬭	⬭
Problèmes d'approvisionnement en TDR empêchant les prestataires de faire les tests	⬭	⬭	⬭
Réticence des pharmaciens / personnel des parapharmacies à effectuer des tests sur des échantillons de sang	⬭	⬭	⬭
Les agents de santé mettent en doute la précision du test (microscopie ou TDR)	⬭	⬭	⬭
L'attitude des agents de santé : pas concernés par la qualité des tests ou de la prise en charge	⬭	⬭	⬭
Difficultés pour prélever les échantillons chez les patients	⬭	⬭	⬭

8. **Autre (veuillez spécifier) :**

____

Skip to question 19.

TESTS DIAGNOSTIQUES POUR LE PALUDISME : OBJECTIFS ET DÉFIS DU PROGRAMME

9. **3.1.5. En vous plaçant du point de vue des patients, dans quelle mesure les facteurs ci-dessous gênent-ils la réalisation des objectifs du programme national en termes de diagnostic et de traitement du paludisme ? Veuillez sélectionner une seule réponse pour chaque rangée.** *

Mark only one oval per row.

	Pas du tout	Un peu	Beaucoup
Refus du prélèvement de sang pour des raisons culturelles, religieuses ou traditionnelles			
Les enfants préfèrent éviter la piqûre au doigt			
Les adultes préfèrent éviter la piqûre au doigt			
Les personnes ne veulent pas se faire tester à cause du délai d'attente très long des résultats			
Les patients veulent recevoir un traitement antipaludique, quels que soient les résultats du test			
Il n'y a pas assez de points de service proposant les tests diagnostiques à proximité des populations qui ont besoin d'être testées			
Commodité du test : les prestataires qui proposent le test ont des horaires d'ouverture peu pratiques et/ou ils sont difficiles d'accès pour des raisons de distance ou de transport			
Les coûts directs et indirects des tests sont perçus comme étant trop élevés (par ex. heures de travail perdues)			
Les patients pensent que l'échantillon de sang est prélevé pour un dépistage du VIH et non du paludisme			
Les patients préfèrent l'automédication			
Pour des raisons religieuses, culturelles ou traditionnelles, les patients ne consultent pas les prestataires qui proposent les tests de dépistage			
Les patients mettent en doute la précision et la qualité de la microscopie ou des TDR			
Les ruptures de stock de tests et de traitements étant fréquentes chez les prestataires qui proposent les tests de diagnostic, les patients tendent à les éviter			

10. **Autre (veuillez spécifier) :**

___

Skip to question 19.

TESTS DIAGNOSTIQUES POUR LE PALUDISME : OBJECTIFS ET DÉFIS DU PROGRAMME

11. **3.2.1. Quels obstacles pourraient empêcher la réalisation des objectifs du programme en termes de diagnostic ? Veuillez sélectionner les propositions les plus importantes, selon vous, dans la liste ci-dessous (maximum trois).** *

Check all that apply.

- [ ] Tout type de problème lié à la microscopie (formation du personnel, qualité, chaîne d'approvisionnement, etc.)
- [ ] Problèmes liés aux produits de diagnostic rapide (par ex. capacité à détecter des infections asymptomatiques, délétion du gène codant pour la protéine HRP2, détermination de l'espèce, etc.)
- [ ] Défis liés au maintien de la qualité des TDR pour le paludisme (par ex. défis liés à la formation sur le changement de TDR, les roulements de personnel, tests effectués sans respecter les POS ou mauvaise interprétation des résultats)
- [ ] Ruptures de stock de TDR et problèmes liés à la chaîne d'approvisionnement
- [ ] Couverture : nombre insuffisant de points de service situés à proximité des patients (par ex. pas assez d'agents de santé communautaire ou de postes de santé dans les zones rurales effectuant des TDR, etc.)
- [ ] Défis liés à la prise en charge par les agents de santé (par ex. directives non suivies – tous les cas suspects ne sont pas testés, les résultats du test de dépistage du paludisme ne sont pas acceptés, ils ne savent pas comment gérer les TDR négatifs)
- [ ] Le degré d'acceptation des tests et des résultats des tests est trop faible
- [ ] Les patients qui ne présentent pas de symptômes hésitent à se faire tester – par ex. pour la recherche réactive de cas ou pour une enquête
- [ ] De nombreux patients consultent des prestataires de soins qui ne proposent pas de test de dépistage (par ex. secteur privé informel, prestataire alternatif/traditionnel)
- [ ] Défis liés au financement de la recherche active de cas et des activités de surveillance
- [ ] Défis liés au financement des tests (fournitures, formation, qualité, etc.)
- [ ] Défis liés aux tests sur échantillon de sang (par ex. sécurité lors de la manipulation du sang et des objets tranchants, élimination des déchets)
- [ ] Problèmes liés à la notification des résultats des tests, compilation des données de surveillance
- [ ] Other: ____________________

Skip to question 19.

TESTS DIAGNOSTIQUES POUR LE PALUDISME : OBJECTIFS ET DÉFIS DU PROGRAMME

12. **3.3.1. Environ quel pourcentage de patients dans votre pays consultent des agents de santé communautaire ? (Si vous ne connaissez pas la réponse, donner votre meilleure estimation)** *

Mark only one oval.

- ( ) 0-5% des patients
- ( ) 6-10% des patients
- ( ) 11-15% des patients
- ( ) 16-20% des patients
- ( ) >21% des patients

13. **3.3.2. Dans certains pays, les secteurs privés formel et de détail effectuent les tests diagnostiques pour le paludisme. Estimez au mieux le nombre de tests (TDR et microscopie) effectués chaque année par le secteur privé dans votre pays.** *
Mark only one oval per row.

	Aucun test effectué	1-100.000 tests	101.000-999.999 tests	1 million- 4,9 millions de tests	5 millions – 20 millions de tests	>20 millions de tests
Secteur privé formel (par ex. hôpitaux, cliniques et laboratoires privés)	◯	◯	◯	◯	◯	◯
Secteur privé de détail (par ex. pharmacies, parapharmacies, etc.)	◯	◯	◯	◯	◯	◯

14. **3.3.3. Quels obstacles pourraient empêcher la réalisation des objectifs du programme en termes de dépistage ? Veuillez sélectionner les propositions les plus importantes, selon vous, dans la liste ci-dessous (maximum trois).** *
Check all that apply.

- [ ] Tout type de problème lié à la microscopie (formation du personnel, qualité, chaîne d'approvisionnement, etc.)
- [ ] Problèmes liés aux produits de diagnostic rapide (par ex. capacité à détecter des infections asymptomatiques, délétion du gène codant pour la protéine HRP2, détermination de l'espèce, etc.)
- [ ] Défis liés au maintien de la qualité des TDR pour le paludisme (par ex. défis liés à la formation sur le changement de TDR, les roulements de personnel, POS non respectées ou mauvaise interprétation des résultats)
- [ ] Ruptures de stock de TDR et problèmes liés à la chaîne d'approvisionnement
- [ ] Couverture : nombre insuffisant de points de service à proximité des patients (par ex. pas assez d'agents de santé communautaire ou de postes de santé dans les zones rurales effectuant des TDR, etc.)
- [ ] Défis liés à la prise en charge par les agents de santé (par ex. directives non suivies – tous les cas suspects ne sont pas testés, les résultats du test de dépistage du paludisme ne sont pas acceptés, ils ne savent pas comment gérer les TDR négatifs)
- [ ] Le degré d'acceptation des tests et des résultats des tests est trop faible
- [ ] Les patients qui ne présentent pas de symptômes hésitent à se faire tester – par ex. pour la recherche réactive de cas ou pour une enquête.
- [ ] De nombreux patients consultent des prestataires de soins qui ne proposent pas de test de dépistage (par ex. secteur privé informel, prestataire alternatif/traditionnel)
- [ ] Défis liés au financement du dépistage actif et des activités de surveillance
- [ ] Défis liés au financement des tests (fournitures, formation, qualité, etc.)
- [ ] Défis liés aux tests sur échantillon de sang (par ex. sécurité lors de la manipulation du sang et des objets tranchants, élimination des déchets)
- [ ] Problèmes liés à la notification des résultats des tests, compilation des données de surveillance
- [ ] Other: ______________________

15. **3.3.4. En vous plaçant du point de vue des agents de santé dans les secteurs public et privé (par ex. médecins, infirmiers, agents de santé communautaire, pharmaciens), dans quelle mesure les facteurs ci-dessous gênent-ils la réalisation des objectifs du programme national en termes de diagnostic et de traitement du paludisme ? Veuillez sélectionner une seule réponse pour chaque rangée.** *

Mark only one oval per row.

	Pas du tout	Un peu	Beaucoup
Sentiment que le test prend trop temps étant donné la charge de travail	⬭	⬭	⬭
Il n'y a pas assez de personnes qualifiées pour effectuer les TDR ou la microscopie	⬭	⬭	⬭
Réticence à appliquer les résultats des tests dans la prise en charge (par ex. patient exigeant un traitement, peur de passer à côté d'une infection dangereuse, confiance plus importante dans l'appréciation clinique /expérience que dans les résultats du test)	⬭	⬭	⬭
Problèmes d'approvisionnement en TDR empêchant les prestataires de faire les tests	⬭	⬭	⬭
Réticence des pharmaciens / personnel des parapharmacies à effectuer des tests sur des échantillons de sang	⬭	⬭	⬭
Les agents de santé mettent en doute la précision du test (microscopie ou TDR)	⬭	⬭	⬭
Attitude des agents de santé : pas concernés par la qualité des tests ou de la prise en charge	⬭	⬭	⬭
Difficulté pour prélever les échantillons chez les patients	⬭	⬭	⬭

16. **Autre (veuillez spécifier) :**

____

17. **3.3.5. En vous plaçant du point de vue des patients, dans quelle mesure les facteurs ci-dessous gênent-ils la réalisation des objectifs du programme national en termes de diagnostic et de traitement du paludisme ? Veuillez sélectionner une seule réponse pour chaque rangée.** *

Mark only one oval per row.

	Pas du tout	Un peu	Beaucoup
Refus de la prise de sang pour des raisons culturelles, religieuses, traditionnelles	◯	◯	◯
Les enfants préfèrent éviter la piqûre au doigt	◯	◯	◯
Les adultes préfèrent éviter la piqûre au doigt	◯	◯	◯
Les personnes ne veulent pas se faire tester à cause du délai d'attente très long des résultats	◯	◯	◯
Les patients veulent recevoir un traitement antipaludique, quels que soient les résultats du test	◯	◯	◯
Il n'y a pas assez de points de service proposant les tests de diagnostic à proximité des populations qui ont besoins d'être testées	◯	◯	◯
Commodité du test : les prestataires qui proposent le test ont des horaires d'ouverture peu pratiques et/ou ils sont difficiles d'accès pour des raisons de distance ou de transport	◯	◯	◯
Les coûts directs et indirects des tests sont perçus comme étant trop élevés (par ex. perte d'heures de travail)	◯	◯	◯
Les patients pensent que l'échantillon de sang est prélevé pour un dépistage du VIH et non du paludisme	◯	◯	◯
Les patients préfèrent l'automédication	◯	◯	◯
Pour des raisons religieuses, culturelles ou traditionnelles, les patients ne consultent pas les prestataires qui proposent les tests diagnostiques	◯	◯	◯
Les patients ne font pas confiance à la précision et à la qualité de la microscopie ou des TDR	◯	◯	◯
Les ruptures de stock de tests et de traitements étant fréquentes parmi les prestataires qui proposent les tests diagnostiques, les patients tendent à les éviter	◯	◯	◯

18. **Autre (veuillez spécifier) :**

____

TESTS DIAGNOSTIQUES POUR LE PALUDISME : OBJECTIFS ET DÉFIS DU PROGRAMME

19. **Est-ce que votre programme national a défini des objectifs et des priorités spécifiques pour le diagnostic du paludisme, ou des domaines prioritaires, actuels ou prévus dans un avenir proche ? Veuillez specifier:**

SCENARIOS D'UTILISATIONS POTENTIELLES ET POPULATIONS CLÉS VISÉES PAR LES TESTS NON INVASIFS

Dans cette section, on vous demande votre avis sur les utilisations potentielles des tests non invasifs ainsi que sur les préférences des utilisateurs pour le prélèvement de sang, d'urine ou de salive.

20. **4. Dans votre pays, les tests non invasifs effectués sur des échantillons de salive ou d'urine pourraient être utiles dans quelles situations ?** *

Mark only one oval per row.

	Certainement pas utile	Probablement pas utile	Probablement utile	Certainement utile	Pas applicable – ce type de test ne serait pas effectué dans ce pays
Test diagnostique effectué dans des structures sanitaires	◯	◯	◯	◯	◯
Test diagnostique effectué par des agents de santé communautaire	◯	◯	◯	◯	◯
Test diagnostique effectué par le secteur privé de détail (pharmacies, parapharmacies)	◯	◯	◯	◯	◯
Recherche active des cas	◯	◯	◯	◯	◯
Dépistage aux frontières	◯	◯	◯	◯	◯
Recherche active des cas dans des populations ou dans les lieux à risque élevé	◯	◯	◯	◯	◯
Test diagnostique effectué dans le cadre d'enquêtes et d'activités de surveillance	◯	◯	◯	◯	◯
Autotest (par ex. kits de test à domicile)	◯	◯	◯	◯	◯
Test diagnostique effectué pendant les flambées de maladies à risque élevé (par ex. Ebola)	◯	◯	◯	◯	◯
Dépistage des femmes enceintes	◯	◯	◯	◯	◯
Test diagnostique effectué chez les enfants	◯	◯	◯	◯	◯

21. **Autre (veuillez spécifier)**

____

22. **4.1. Si vous avez sélectionné l'autotest, veuillez décrire les groupes de population et les contextes qui bénéficieraient de l'autotest. Par exemple, est-ce que les kits de test seraient mis à la disposition du grand public (par ex. par le biais des installations de santé publique pour que les personnes puissent les emmener chez eux), ou est-ce qu'ils seraient vendus dans des parapharmacies ou des pharmacies, et est-ce que l'autotest serait limité à certaines populations spécifiques difficiles d'accès, aux voyageurs, etc. ?**

23. **5. D'après vous, quelle serait la réaction des populations suivantes à un test diagnostique sur échantillon de salive s'il existait aujourd'hui ?** *
Mark only one oval per row.

	Test salivaire facilement accepté	Hésitation possible au début pour passer au test salivaire, mais acceptation ensuite	Préférence pour le test sanguin, peu de chances d'utiliser le test salivaire	Pas applicable dans notre pays
Enfants	◯	◯	◯	◯
Adultes	◯	◯	◯	◯
Migrants/ populations mobiles	◯	◯	◯	◯
Populations autochtones	◯	◯	◯	◯
Communautés rurales/retirées	◯	◯	◯	◯
Populations testées dans le cadre d'une enquête (généralement asymptomatiques) dans un but de surveillance	◯	◯	◯	◯
Professionnels de santé (par ex. médecins, infirmiers)	◯	◯	◯	◯
Agents de santé communautaire	◯	◯	◯	◯
Détaillants (vendeurs dans les parapharmacies, pharmaciens)	◯	◯	◯	◯

24. **6. D'après vous, quelle serait la réaction des populations suivantes à un test diagnostique sur échantillon d'urine s'il existait aujourd'hui ?** *

Mark only one oval per row.

	facilement accepté	passer au test urinaire, mais acceptation ensuite	peu de chances d'utiliser le test urinaire	Pas applicable dans notre pays
Enfants	◯	◯	◯	◯
Adultes	◯	◯	◯	◯
Migrants/ populations mobiles	◯	◯	◯	◯
Populations autochtones	◯	◯	◯	◯
Communautés rurales/retirées	◯	◯	◯	◯
Populations testées dans le cadre d'une enquête (généralement asymptomatiques) dans un but de surveillance	◯	◯	◯	◯
Professionnels de santé (par ex. médecins, infirmiers)	◯	◯	◯	◯
Agents de santé communautaire	◯	◯	◯	◯
Détaillants (vendeurs dans les parapharmacies, pharmaciens)	◯	◯	◯	◯
Enfants	◯	◯	◯	◯

25. **7. D'après vous, quelles sont les croyances culturelles concernant les prélèvements de sang, quelles seraient les attentes des patients concernant les tests sanguins et le paludisme dans votre pays, et quel serait l'impact de ces facteurs sur les préférences des patients pour les tests diagnostiques pour le paludisme sur échantillon de sang ou non invasifs ?**

____
____
____
____
____

SCENARIOS D'UTILISATIONS POTENTIELLES ET POPULATIONS CLÉS VISÉES PAR LES TESTS NON INVASIFS

26. **8. En supposant que les performances des tests soient équivalentes, quel serait le premier choix de votre programme pour le type de prélèvement utilisé pour le test diagnostique pour le paludisme ?** *

Mark only one oval.

◯ Urinaire
◯ Salivaire
◯ Sanguin

27. **9. En supposant que les performances des tests soient équivalentes, quel serait le deuxième choix de votre programme pour le type de prélèvement utilisé pour le test diagnostique pour le paludisme ?** *

Mark only one oval.

- Urinaire
- Salivaire
- Sanguin

28. **10. Les énoncés ci-dessous décrivent l'impact des tests non invasifs sur la santé publique de votre pays. Veuillez indiquer dans quelle mesure vous êtes d'accord ou pas avec ces énoncés :** *

Mark only one oval per row.

	Pas du tout d'accord	Pas d'accord	D'accord	Tout à fait d'accord	Pas applicable à notre pays
Le test diagnostique pour le paludisme étant déjà très peu invasif, bien accepté et couramment effectué dans notre pays, il n'est pas nécessaire de le changer pour des tests non invasifs sauf si les nouveaux tests présentent d'autres avantages (par ex. performance significativement améliorée, délai plus court pour obtenir les résultats, etc.)	◯	◯	◯	◯	◯
Les tests non invasifs pourraient améliorer significativement l'accès au diagnostic pour les populations difficiles à atteindre	◯	◯	◯	◯	◯
Les tests non invasifs pourraient améliorer significativement l'accès au diagnostic dans l'ensemble du pays	◯	◯	◯	◯	◯
Élargir le nombre de personnes pouvant effectuer le test est une mesure importante pour notre programme	◯	◯	◯	◯	◯
Les tests non invasifs pourraient augmenter le nombre de tests effectués dans le secteur privé de détail	◯	◯	◯	◯	◯
L'autotest (par ex. test effectué à la maison) pourrait permettre aux patients de consulter plus tôt les professionnels de santé en cas de paludisme	◯	◯	◯	◯	◯
Le test sanguin pour le diagnostic du paludisme ne présente aucun problème dans notre pays	◯	◯	◯	◯	◯

TEST SALIVAIRE : ADOPTION ET PRIX

Pour cette section, veuillez considérer que le test salivaire :

• Est aussi sensible que le TDR traditionnel pour le diagnostic du paludisme, mais qu'il est en plus capable de détecter les parasites avec des délétions du gène codant pour la protéine HRP2, et sa corrélation avec l'infection active est bonne.

• Est facile à transporter et à utiliser (par ex. jetable, nécessite une formation minimale des opérateurs ou le test comporte peu d'étapes), et les résultats sont obtenus en <30 minutes.

- Est capable de diagnostiquer un, deux, ou une combinaison d'antigènes (résultat avec une ou deux lignes), en fonction de votre pays.
- Fournit un résultat qualitatif.

29. **11. En supposant que le prix ne soit pas un problème et que ce type de test salivaire existe actuellement, quelle serait la probabilité que votre programme utilise ce test ?** *

Mark only one oval.

- ( ) Extrêmement probable *Skip to question 30.*
- ( ) Très probable *Skip to question 30.*
- ( ) Peu probable *Skip to question 31.*
- ( ) Improbable *Skip to question 31.*

TEST SALIVAIRE : ADOPTION ET PRIX

30. **11.1. Si le test salivaire était disponible, est-ce que votre programme envisagerait de mener de nouvelles activités ? Parmi la liste ci-dessous, veuillez sélectionner les activités que votre programme pourrait effectuer si un test salivaire était disponible :** *

Check all that apply.

- [ ] Test diagnostique effectué par des agents de santé communautaire
- [ ] Test diagnostique effectué par le secteur privé de détail (par ex. parapharmacies et pharmacies)
- [ ] Recherche réactive des cas
- [ ] Dépistage aux frontières
- [ ] Recherche proactive des cas
- [ ] Enquêtes/activités de surveillance supplémentaires nécessitant des tests
- [ ] Autotest (par ex. kits de test à domicile accessible au grand public)
- [ ] Autotest limité (par ex. fournis par le secteur privé pour les populations difficiles d'accès, les personnes vivant dans des zones retirées, ou la population générale pendant les flambées de maladies à risque élevé)
- [ ] Kits d'autotest pour les voyageurs
- [ ] Test diagnostique effectué par les agents de santé pendant les flambées de maladies à risque élevé (par ex. Ebola)
- [ ] Other: ____________________

Skip to question 32.

TEST SALIVAIRE : ADOPTION ET PRIX

31. **11.2. Quelle est la raison principale pour laquelle le programme a peu de chances d'utiliser un test salivaire non invasif ? Sélectionnez la réponse qui convient le mieux.** *

Mark only one oval.

- Nous sommes généralement satisfaits des tests diagnostiques actuels
- Manque général de confiance dans les tests salivaires
- Les prestataires ont peu de chances de préférer ce test aux tests sanguins
- Les patients ont peu de chances de préférer ce test aux tests sanguins
- Pour tout nouveau test diagnostique pour le paludisme, nous préférons des tests avec plus de deux lignes pour la détermination des espèces
- Pour tout nouveau test diagnostique pour le paludisme, nous préférons des résultats quantitatifs
- Pour tout nouveau test diagnostique pour le paludisme, nous préférons des tests capables de détecter les infections asymptomatiques
- Nous pensons que le test salivaire sera plus cher que les tests actuels, et qu'il ne sera donc pas abordable pour le programme
- Other: ____________________

TEST SALIVAIRE : ADOPTION ET PRIX

32. **12. Veuillez indiquer comment votre programme serait susceptible d'utiliser le test salivaire aux prix suivants : ** Cette question est destinée à nous aider à mieux comprendre les facteurs influençant l'adoption et l'utilisation du test à différents prix. Nous savons qu'il n'est pas facile d'y répondre, nous vous demandons seulement votre meilleure estimation. **** *

Mark only one oval per row.

	Probablement non utilisé	Utilisation très limitée (par ex. situations ou populations spécifiques)	Remplacerait partiellement certains tests utilisés actuellement	Remplacerait largement les TDR utilisés actuellement	Remplacerait largement les TDR et la microscopie utilisés actuellement
0,0-0,50 USD	○	○	○	○	○
0,51-1,00 USD	○	○	○	○	○
1,01-1,50 USD	○	○	○	○	○
>1,51 USD	○	○	○	○	○

33. **13. Supposez maintenant que ce test urinaire soit plus sensible que le TDR traditionnel et qu'il soit capable de détecter des infections inframicroscopiques** *

Mark only one oval per row.

	Probablement non utilisé	Utilisation très limitée (par ex. situations ou populations spécifiques)	Remplacerait partiellement certains tests utilisés actuellement	Remplacerait largement les TDR utilisés actuellement	Remplacerait largement les TDR et la microscopie utilisés actuellement
0,51-1,00 USD	○	○	○	○	○
1,01-1,50 USD	○	○	○	○	○
>1,51 USD	○	○	○	○	○

ADOPTION ET PRIX DU TEST URINAIRE

Pour cette section, veuillez considérer que le test urinaire pour le diagnostic du paludisme :

- Est aussi sensible que le TDR traditionnel pour le paludisme, mais qu'il est en plus capable de

détecter les parasites avec des délétions du gène codant pour la protéine HRP2, et sa corrélation avec l'infection active est bonne.

- Est facile à transporter et à utiliser (par ex. jetable, nécessite une formation minimale des opérateurs ou peu d'étapes pour la réalisation du test), et les résultats sont obtenus en <30 minutes.
- Peut inclure une ou deux lignes en fonction de votre pays.
- Fournit un résultat qualitatif.

34. **14. En supposant que le prix ne soit pas un problème et que ce type de test urinaire existe actuellement, quelle serait la probabilité que votre programme utilise ce test ?** *

Mark only one oval.

- ( ) Extrêmement probable *Skip to question 35.*
- ( ) Très probable *Skip to question 35.*
- ( ) Peu probable *Skip to question 36.*
- ( ) Pas du tout probable *Skip to question 36.*

ADOPTION ET PRIX DU TEST URINAIRE

35. **14.1. Si le test urinaire était disponible, est-ce que votre programme envisagerait de mener de nouvelles activités ? Parmi la liste ci-dessous, veuillez sélectionner les activités que votre programme pourrait effectuer si un test urinaire était disponible :** *

Check all that apply.

- [ ] Test diagnostique du paludisme effectué par des agents de santé communautaire
- [ ] Test diagnostique du paludisme effectué par le secteur privé de détail (par ex. parapharmacies et pharmacies)
- [ ] Recherche réactive des cas
- [ ] Dépistage aux frontières
- [ ] Recherche proactive des cas
- [ ] Enquêtes/activités de surveillance supplémentaires nécessitant des tests
- [ ] Autotest (par ex. kit de test à domicile accessible au grand public)
- [ ] Autotest limité (par ex. fournis par le secteur privé uniquement pour les populations difficiles d'accès, les personnes vivant dans des zones retirées, ou la population générale pendant les flambées de maladies à risque élevé)
- [ ] Kit d'autotest pour les voyageurs
- [ ] Test diagnostique du paludisme effectué par les agents de santé pendant les flambées de maladies à risque élevé (par ex. Ebola)
- [ ] Other: ____________________

Skip to question 37.

ADOPTION ET PRIX DU TEST URINAIRE

36. **14.2. Quelle est la raison principale pour laquelle le programme a peu de chances d'utiliser un test urinaire non invasif ? Veuillez choisir la réponse qui convient le mieux.** *
Mark only one oval.

- Nous sommes généralement satisfaits des tests diagnostiques actuels
- Manque général de confiance dans les tests urinaires
- Les prestataires ont peu de chances de préférer ce test aux tests sanguins
- Les patients ont peu de chances de préférer ce test aux tests sanguins
- Pour tout nouveau test de diagnostic du paludisme, nous préférons des tests avec plus de deux lignes pour la détermination des espèces
- Pour tout nouveau test de diagnostic du paludisme, nous préférons des résultats quantitatifs
- Pour tout nouveau test de diagnostic du paludisme, nous préférons des tests capables de détecter les infections asymptomatiques
- Nous pensons que le test urinaire sera plus cher que les tests actuels, et qu'il ne sera donc pas abordable pour le programme
- Other: ______

ADOPTION ET PRIX DU TEST URINAIRE

37. **15. Veuillez indiquer comment votre programme serait susceptible d'utiliser le test salivaire aux prix suivants : *** Cette question est destinée à nous aider à mieux comprendre les facteurs influençant l'adoption et l'utilisation du test à différents prix. Nous savons qu'il n'est pas facile d'y répondre, nous vous demandons seulement votre meilleure estimation. **** *
Mark only one oval per row.

	Probablement non utilisé	Utilisation très limitée (par ex. situations ou populations spécifiques)	Remplacerait partiellement certains tests utilisés actuellement	Remplacerait largement les TDR utilisés actuellement	Remplacerait largement les TDR et la microscopie utilisés actuellement
0,0-0,50 USD					
0,51-1,00 USD					
1,01-1,50 USD					
>1,51 USD					

38. **16. Supposez maintenant que ce test urinaire soit plus sensible que le TDR traditionnel et qu'il soit capable de détecter des infections inframicroscopiques** *
Mark only one oval per row.

	Probablement non utilisé	Utilisation très limitée (par ex. situations ou populations spécifiques)	Remplacerait partiellement certains tests utilisés actuellement	Remplacerait largement les TDR utilisés actuellement	Remplacerait largement les TDR et la microscopie utilisés actuellement
0,51-1,00 USD					
1,01-1,50 USD					
>1,51 USD					

PRÉFÉRENCES POUR LES TESTS NON INVASIFS

39. **17. Il existe toutes une variété de types de tests possibles (c'est à dire capables de détecter différentes combinaisons d'antigènes). Supposez qu'il existe un test non invasif avec deux lignes. Quelle association d'antigènes sera la plus utile pour votre programme ? * Supposez que Pf détecte tous les types de parasites P. falciparum, même en cas de délétions du gène codant pour la protéine HRP2.** *

Mark only one oval.

- Pan/Pf
- Pf/Pv
- Pan/Pv
- Pan/Pvo
- Pf/Pvo
- Other: ____________

40. **18. Parmi la liste ci-dessous, veuillez sélectionner les préférences de votre programme concernant les caractéristiques recherchées pour un nouveau test diagnostique pour le paludisme :** *

Mark only one oval per row.

	Absolument essentiel	Utile, optimal	Probablement pas nécessaire
Stable à 40C			
Durée de conservation 18 mois			
Durée de conservation 24 mois			
Délai d'obtention des résultats plus court que les TDR actuels			
Capable de détecter des infections inframicroscopiques			
Résultat quantitatif			
Utilisation possible par des personnes non formées (avec un simple mode d'emploi sur base de pictogrammes)			

41. **19. Cette question concerne l'utilisation de lecteurs, petits appareils portables à piles, disposant d'une autonomie d'environ 24 heures. Les lecteurs peuvent effectuer plusieurs fonctions : i) augmenter la sensibilité du TDR ; ii) automatiser la saisie des données (utilisation du test, résultats, coordonnées GPS) ainsi que le transfert des données vers une base de données centrale disposant d'un logiciel d'analyse ; et iii) améliorer la qualité en guidant l'utilisateur avec un mode d'emploi et en interprétant les résultats. Les inconvénients sont un coût plus élevé et peut être un accès plus restreint à cause de l'appareil supplémentaire. Quelle serait la probabilité que votre programme utilise un test non invasif muni d'un lecteur?** *

Mark only one oval.

- Extrêmement probable
- Très probable
- Peu probable
- Pas du tout probable

FIN DE L'ENQUÊTE

Nous vous remercions pour votre participation à cette enquête et pour avoir pris le temps de répondre le mieux possible à nos questions. Vos commentaires sont extrêmement précieux pour cette initiative, dont le but est de nous aider à prendre des décisions éclairées concernant le développement de nouveaux outils diagnostiques et l'amélioration de la prise en charge des cas, de la surveillance, et de

l’élimination du paludisme. Ces informations seront aussi utiles à d’autres organisations travaillant sur le paludisme ainsi qu’aux donateurs.

42. **Si vous voulez apporter des commentaires supplémentaires, n’hésitez pas à les noter dans l’encadré ci-dessous.**

43. **Nous sommes aussi en train d’élaborer une autre petite enquête destinée à recueillir les avis d’agents de santé sur les tests sanguins, salivaires et urinaires. Nous vous serions très reconnaissants si vous pouviez nous fournir le nom et l’adresse email de quelques agents de santé dans votre pays susceptibles de bien vouloir participer à cette enquête. Nous recherchons surtout des infirmiers, des personnes travaillant pour des programmes de prise en charge intégrée des maladies de l'enfant (IMCI), des agents de santé communautaire, et des prestataires travaillant dans des établissements locaux du secteur privé.**

Powered by
Google Forms
