# Supplementary material for: Acceptance and perceived value of non-invasive malaria diagnostic tests in malaria-endemic countries
Source: Malar J. 2021 Sep 24;20:379. doi: 10.1186/s12936-021-03911-y (PMC8461884; doi:10.1186/s12936-021-03911-y)
Supplement: Supplementary file 3 — Additional file 3. Structured questionnaire in Spanish. [file 12936_2021_3911_MOESM3_ESM.pdf]

ENCUESTA SOBRE PRUEBAS DE DIAGNÓSTICO NO INVASIVAS PARA MALARIA

* Required

FIND Because diagnosis matters

Gracias por participar en la encuesta. Por favor, presione "SIGUIENTE" para empezar

INTRODUCCIÓN E INSTRUCCIONES

La fundación Bill and Melinda Gates está considerando apoyar el desarrollo de pruebas de diagnóstico no invasivas para la detección de malaria. Estos tests serían similares a las pruebas de diagnóstico rápidas (PDRs) ya existentes, pero utilizarían saliva u orina como muestra, en vez de sangre. Antes de invertir en el desarrollo de estas herramientas de diagnóstico, la fundación nos ha pedido ayudarles a entender el mercado para estas pruebas no invasivas, así como estimar cualquier impacto potencial que estas pudieran tener en el control y eliminación de la malaria a nivel global.

En relación a esto, nos gustaría conocer su opinión sobre diferentes aspectos:

I. Prioridades en su programa de malaria y los retos relacionados con las pruebas de diagnóstico
II. Posibles escenarios o poblaciones en los que se podrían aplicar estas pruebas no invasivas
III. Adopción y coste
IV. Preferencias en cuanto a las características fundamentales del producto

Cuando conteste a estas preguntas, por favor, considere su país y la experiencia de su programa nacional. Sus respuestas serán analizadas conjuntamente junto a las de otros participantes; no evaluaremos respuestas individuales. Por lo tanto, le pedimos que sea lo más honesto posible. No existen respuestas correctas o incorrectas. Reconocemos que es difícil proporcionar el punto de vista para un producto que todavía no existe y para el cual quizá no conozca las respuestas a distintas preguntas de la encuesta. No obstante, estamos buscando suposiciones informadas y predicciones basadas en su experiencia.

El tiempo estimado para contestar todas las preguntas es de 20 minutos. ¡Muchas gracias de antemano por participar!

DATOS DEMOGRÁFICOS

La encuesta es anónima, pero nos gustaría analizar los datos por región e institución (por ejemplo, respuesta del programa de malaria nacional).

1. **1. ¿En qué país trabaja actualmente? (Si trabaja en diferentes países, por favor, seleccione el país en el que pasa la mayor parte del tiempo trabajando)** *

Mark only one oval.

- Afghanistan
- Albania
- Algeria
- American Samoa
- Andorra
- Angola
- Anguilla
- Antigua & Barbuda
- Argentina
- Armenia
- Aruba
- Australia
- Austria
- Azerbaijan
- Bahamas, The
- Bahrain
- Bangladesh
- Barbados
- Belarus
- Belgium
- Belize
- Benin
- Bermuda
- Bhutan
- Bolivia
- Bosnia & Herzegovina
- Botswana
- Brazil
- British Virgin Is.
- Brunei
- Bulgaria
- Burkina Faso
- Burma
- Burundi
- Cambodia
- Cameroon
- Canada
- Cape Verde
- Cayman Islands
- Central African Rep.

- Chad
- Chile
- China
- Colombia
- Comoros
- Congo, Dem. Rep.
- Congo, Repub. of the
- Cook Islands
- Costa Rica
- Cote d'Ivoire
- Croatia
- Cuba
- Cyprus
- Czech Republic
- Denmark
- Djibouti
- Dominica
- Dominican Republic
- East Timor
- Ecuador
- Egypt
- El Salvador
- Equatorial Guinea
- Eritrea
- Estonia
- Ethiopia
- Faroe Islands
- Fiji
- Finland
- France
- French Guiana
- French Polynesia
- Gabon
- Gambia, The
- Gaza Strip
- Georgia
- Germany
- Ghana
- Gibraltar
- Greece
- Greenland
- Grenada

- Guadeloupe
- Guam
- Guatemala
- Guernsey
- Guinea
- Guinea-Bissau
- Guyana
- Haiti
- Honduras
- Hong Kong
- Hungary
- Iceland
- India
- Indonesia
- Iran
- Iraq
- Ireland
- Isle of Man
- Israel
- Italy
- Jamaica
- Japan
- Jersey
- Jordan
- Kazakhstan
- Kenya
- Kiribati
- Korea, North
- Korea, South
- Kuwait
- Kyrgyzstan
- Laos
- Latvia
- Lebanon
- Lesotho
- Liberia
- Libya
- Liechtenstein
- Lithuania
- Luxembourg
- Macau
- Macedonia

- Madagascar
- Malawi
- Malaysia
- Maldives
- Mali
- Malta
- Marshall Islands
- Martinique
- Mauritania
- Mauritius
- Mayotte
- Mexico
- Micronesia, Fed. St.
- Moldova
- Monaco
- Mongolia
- Montserrat
- Morocco
- Mozambique
- Namibia
- Nauru
- Nepal
- Netherlands
- Netherlands Antilles
- New Caledonia
- New Zealand
- Nicaragua
- Niger
- Nigeria
- N. Mariana Islands
- Norway
- Oman
- Pakistan
- Palau
- Panama
- Papua New Guinea
- Paraguay
- Peru
- Philippines
- Poland
- Portugal
- Puerto Rico

- Qatar
- Reunion
- Romania
- Russia
- Rwanda
- Saint Helena
- Saint Kitts & Nevis
- Saint Lucia
- St Pierre & Miquelon
- Saint Vincent and the Grenadines
- Samoa
- San Marino
- Sao Tome & Principe
- Saudi Arabia
- Senegal
- Serbia
- Seychelles
- Sierra Leone
- Singapore
- Slovakia
- Slovenia
- Solomon Islands
- Somalia
- South Africa
- Spain
- Sri Lanka
- Sudan
- Suriname
- Swaziland
- Sweden
- Switzerland
- Syria
- Taiwan
- Tajikistan
- Tanzania
- Thailand
- Togo
- Tonga
- Trinidad & Tobago
- Tunisia
- Turkey
- Turkmenistan

- Turks & Caicos Is
- Tuvalu
- Uganda
- Ukraine
- United Arab Emirates
- United Kingdom
- United States
- Uruguay
- Uzbekistan
- Vanuatu
- Venezuela
- Vietnam
- Virgin Islands
- Wallis and Futuna
- West Bank
- Western Sahara
- Yemen
- Zambia
- Zimbabwe

2. **2. ¿Para qué tipo de institución trabaja?** *

Check all that apply.

- [ ] Programa nacional de control de malaria
- [ ] Ministerio de salud
- [ ] Programa de control de enfermedades transmitidas por vectores
- [ ] Centro nacional para el control de enfermedades y salud pública
- [ ] El programa de malaria del Fondo Mundial
- [ ] Oficina local o regional de la Organización Mundial de la Salud (OMS)
- [ ] Organización Mundial de la Salud (OMS)
- [ ] Universidad / Institución educativa
- [ ] Other: ____________________

DIAGNÓSTICO DE MALARIA: OBJETIVOS Y RETOS DE SU PROGRAMA

3. **3. El diagnóstico de malaria es importante para el manejo de casos, así como para la vigilancia de esta enfermedad y las actividades de eliminación. Actualmente, ¿cuáles de las siguientes afirmaciones reflejan mejor los objetivos de su programa nacional?** *

Mark only one oval.

- ( ) 3.1. El programa está principalmente centrado en incrementar la tasa de pruebas que se realizan, diagnosticar al máximo número de persona con fiebre sospechosas de malaria y tratarlas apropiadamente (i.e. expandir el acceso a un manejo de casos de calidad). *Skip to question 4.*
- ( ) 3.2. El programa está principalmente centrado en realizar pruebas para la eliminación de malaria o para prevenir su reintroducción, tales como vigilancia de la enfermedad o disminución del reservorio identificando focos de infección, evitando nuevos brotes. *Skip to question 11.*
- ( ) 3.3. El programa está centrado en ambos puntos anteriormente (3.1 y 3.2), dependiendo de la región del país. *Skip to question 12.*

DIAGNÓSTICO DE MALARIA: OBJETIVOS Y RETOS DE SU PROGRAMA

4. **3.1.1. Aproximadamente, ¿qué porcentaje de pacientes en su país buscan cuidado médico del personal sanitario de la comunidad? (Si no tiene datos, por favor proporcione una estimación)** *

Mark only one oval.

- ( ) 0-5% de los pacientes
- ( ) 6-10% de los pacientes
- ( ) 11-15% de los pacientes
- ( ) 16-20% de los pacientes
- ( ) >21% de los pacientes

5. **3.1.2. En algunos países, el sector privado, ambos formal (i.e. hospitales privados, clínicas o laboratorios privados) y minorista, realiza pruebas para el diagnóstico de malaria. Por favor, proporcione su mejor estimación sobre el número de pruebas (PDR y microscopía) que el sector privado realiza cada año.** *

Mark only one oval per row.

	No realiza ninguna prueba	1-100.000 pruebas	101.000-999.999 pruebas	1 millón- 4,9 millones pruebas	5 millón – 20 millones pruebas	>20 millones pruebas
Formal sector privado (ej. hospitales privados, clínicas y laboratorios)	( )	( )	( )	( )	( )	( )
Sector privado minorista (ej. farmacias)	( )	( )	( )	( )	( )	( )

6. **3.1.3. ¿Qué podría interferir en el alcance de los objetivos del programa en torno a la realización de pruebas? Por favor, seleccione hasta tres ítems de la lista que considere más importantes** *

Check all that apply.

- [ ] Problemas con la microscopía (por ejemplo, necesidad de entrenar al personal, control de calidad, suministro, etc.)
- [ ] Problemas relacionados con las PDR (por ejemplo, capacidad de detectar infecciones asintomáticas, delecciones del gen HRP2, persistencia de HRP2, etc.)
- [ ] Dificultades manteniendo la calidad de las PDR para malaria (por ejemplo, dificultades relacionadas con el entrenamiento del personal cuando se cambia de PDR, rotación de personal, problemas del personal para seguir los procedimientos estandarizados de trabajo o interpretando los resultados, etc.)
- [ ] Problemas con las existencias de PDRs y su subministro
- [ ] Cobertura: falta de centros o servicios cerca de los pacientes (por ejemplo, insuficientes centros de salud en áreas rurales que realizan PDR o falta de personal sanitario en la comunidad)
- [ ] Dificultades con el manejo de casos por parte del personal sanitario (por ejemplo, el personal sanitario no sigue las guías - fallo en la realización de pruebas en todos los casos sospechosos, falta de aceptación de los resultados de la prueba de malaria, falta de conocimiento para manejar casos negativos por la PDR)
- [ ] La aceptación de la realización de pruebas y de los resultado por parte del paciente es muy baja
- [ ] Muchos pacientes buscan cuidados de proveedores que no ofrecen realizar pruebas (por ejemplo, sector privado minorista, cuidados médicos alternativos tales como remedios botánicos, curanderos)
- [ ] Desafíos relacionados con la financiación para realizar pruebas (suministros, PDR, capacitación, calidad, etc.)
- [ ] Desafíos relacionados con la realización de pruebas basadas en muestras sanguíneas (por ejemplo, manejo seguro de la sangre y de los objetos punzantes, correcta eliminación de desechos)
- [ ] Problemas relacionados con la notificación de los resultados de las pruebas, compilación de los datos de vigilancia
- [ ] Other: ______________________

7. **3.1.4. Por favor, considere el punto de vista del PROFESIONAL SANITARIO en el sector público y privado (por ejemplo, doctores, enfermeras, personal sanitario en la comunidad, farmacéuticos). ¿En qué medida alguno de los siguientes puntos de la tabla limita el logro de los objetivos de su programa nacional para la realización de pruebas diagnósticas y el tratamiento de la malaria? Por favor, seleccione una respuesta por línea:** *
Mark only one oval per row.

	No limitante en absoluto	Algo limitante	Muy limitante
Percepción de que la realización de pruebas diagnósticas lleva demasiado tiempo, dada la carga de trabajo	◯	◯	◯
No hay suficiente personal cualificado para realizar las PDR o la microscopía	◯	◯	◯
Reticencia a la hora de manejar pacientes en función de los resultados de las pruebas (por ejemplo, expectativas del paciente para el tratamiento, no querer perder una infección peligrosa, confianza en la experiencia clínica sobre los resultados de las pruebas)	◯	◯	◯
Suministro inconsistente de PDR que impide que los especialistas clínicos realicen pruebas	◯	◯	◯
Los farmacéuticos / trabajadores de farmacias son reacios a realizar pruebas basadas en sangre	◯	◯	◯
El personal sanitario no confía en la precisión de la pruebas de diagnóstico (microscopía o PDR)	◯	◯	◯
Actitud del personal sanitario: no están preocupados sobre la calidad de la prueba o sobre el manejo de casos	◯	◯	◯
Dificultad para obtener muestras de pacientes	◯	◯	◯

8. **Other (please specify)**

____

Skip to question 19.

DIAGNÓSTICO DE MALARIA: OBJETIVOS Y RETOS DE SU PROGRAMA

9. **3.1.5. Por favor, considere ahora la perspectiva de los PACIENTES, ¿en qué medida alguno de los siguientes puntos de la tabla limita el logro de los objetivos de su programa nacional para la realización de pruebas diagnósticas y el tratamiento de la malaria? Por favor, seleccione una respuesta por línea:** *

Mark only one oval per row.

	No limitante en absoluto	Algo limitante	Muy limitante
Razones culturales, religiosas, tradicionales para evitar la extracción de sangre	◯	◯	◯
Los niños prefieren evitar la punción en el dedo para extraer sangre	◯	◯	◯
Los adultos prefieren evitar la punción en el dedo para extraer sangre	◯	◯	◯
La gente evita realizarse pruebas de diagnóstico debido a los largos tiempos de espera para obtener resultados	◯	◯	◯
Los pacientes esperan un tratamiento anti-malaria, independientemente del resultado de la prueba	◯	◯	◯
No hay suficientes centros de servicio para realizar las pruebas cerca de la población que las necesita	◯	◯	◯
Conveniencia de las pruebas: los especialistas clínicos que realizan las pruebas no están disponibles durante las horas más convenientes y/o no están a una distancia conveniente (hogar/transporte)	◯	◯	◯
El coste de realización de las pruebas se percibe como elevado, tanto los costes directos como indirectos (por ejemplo, pérdida de tiempo laboral)	◯	◯	◯
Los pacientes piensan que la sangre se extrae para realizar la prueba del SIDA y no para malaria	◯	◯	◯
Los pacientes prefieren auto-tratarse/auto-medicarse	◯	◯	◯
Debido a motivos religiosos, culturales o costumbres tradicionales, los pacientes no buscan cuidado médico de los especialistas clínicos que realizan pruebas de diagnostico	◯	◯	◯
El pacientes no confían en la precisión y calidad de la microscopía o de las PDRs	◯	◯	◯
La falta de existencias de pruebas de diagnóstico y tratamiento son comunes entre los especialistas clínicos que pueden realizar estas pruebas, por lo que los pacientes evitan estos centros de salud	◯	◯	◯

10. **Other (please specify)**

____

Skip to question 19.

DIAGNÓSTICO DE MALARIA: OBJETIVOS Y RETOS DE SU PROGRAMA

11. **3.2.1. ¿Qué podría afectar al programa para alcanzar los objetivos en torno a la realización de pruebas de diagnóstico? Seleccione hasta tres ítems de la siguiente lista que considere más importantes.** *

Check all that apply.

- [ ] Problemas con la microscopía (por ejemplo, necesidad de entrenar al personal, control de calidad, suministro, etc.)
- [ ] Problemas relacionados con pruebas de diagnóstico rápidas (por ejemplo, capacidad de detectar infecciones asintomáticas, delecciones del gen HRP2, especiación, etc.)
- [ ] Dificultades manteniendo la calidad de las PDR para malaria (por ejemplo, dificultades relacionadas con el entrenamiento del personal cuando se cambia de PDR, rotación de personal, problemas del personal para seguir los procedimientos estandarizados de trabajo o interpretando los resultados)
- [ ] Problemas con las existencias de PDRs y su subministro
- [ ] Cobertura: falta de centros o servicios cerca de los pacientes (por ejemplo, insuficientes centros de salud en áreas rurales que realizan PDR o falta de personal sanitario en la comunidad)
- [ ] Dificultades con el manejo de casos por parte del personal sanitario (por ejemplo, el personal sanitario no sigue las guías - fallo en la realización de pruebas en todos los casos sospechosos, no aceptación de los resultados de la prueba de malaria, falta de conocimiento para manejar casos negativos por la PDR)
- [ ] La aceptación de la realización de pruebas de diagnóstico y de los resultado por parte del paciente es muy baja
- [ ] Los pacientes que no presentan síntomas son reticentes a que se les realicen las pruebas, por ejemplo, durante la detección reactiva de casos o encuestas poblacionales.
- [ ] Muchos pacientes buscan cuidados de especialistas clínicos que no ofrecen realizar pruebas (por ejemplo, sector privado minorista, cuidados médicos alternativos tales como remedios botánicos, curanderos)
- [ ] Desafíos relacionados con la financiación de la detección activa de casos y las actividades de vigilancia
- [ ] Desafíos relacionados con la financiación de las pruebas de diagnóstico (suministros, entrenamiento, control calidad, etc.)
- [ ] Desafíos relacionados con la realización de pruebas basadas en muestras sanguíneas (por ejemplo, manejo seguro de la sangre y de los objetos punzantes, correcta eliminación de desechos)
- [ ] Problemas relacionados con la notificación de los resultados de las pruebas, compilando datos de vigilancia
- [ ] Other: ____________________

Skip to question 19.

DIAGNÓSTICO DE MALARIA: OBJETIVOS Y RETOS DE SU PROGRAMA

12. **3.3.1. Aproximadamente, ¿qué porcentaje de pacientes en su país buscan cuidado médico del personal sanitario de la comunidad? (Si no tiene datos, por favor proporcione una estimación)** *

Mark only one oval.

- 0-5% de los pacientes
- 6-10% de los pacientes
- 11-15% de los pacientes
- 16-20% de los pacientes
- >21% de los pacientes

13. **3.3.2. En algunos países, el sector privado, ambos formal (i.e. hospitales privados, clínicas o laboratorios privados) y minorista, realiza pruebas para el diagnóstico de malaria. Por favor, proporcione su mejor estimación sobre el número de pruebas (PDR y microscopía) que el sector privado realiza cada año** *

Mark only one oval per row.

	No realiza ninguna prueba	1-100.000 pruebas	101.000-999.999 pruebas	1 millón- 4,9 millones pruebas	5 millón – 20 millones pruebas	>20 millones pruebas
Formal sector privado (ej. hospitales privados, clínicas y laboratorios)						
Sector privado minorista (ej. farmacias)						

14. **3.3.3. ¿Qué podría interferir en el alcance de los objetivos del programa en torno a la realización de pruebas? Por favor, seleccione hasta tres ítems de la lista que considere más importantes** *

Check all that apply.

- [ ] Problemas con la microscopía (por ejemplo, necesidad de entrenar al personal, control de calidad, suministro, etc.)
- [ ] Problemas relacionados con las PDR (por ejemplo, capacidad de detectar infecciones asintomáticas, delecciones del gen HRP2, persistencia de HRP2, etc.)
- [ ] Dificultades manteniendo la calidad de las PDR para malaria (por ejemplo, dificultades relacionadas con el entrenamiento del personal cuando se cambia de PDR, rotación de personal, problemas del personal para seguir los procedimientos estandarizados de trabajo o interpretando los resultados, etc.)
- [ ] Problemas con las existencias de PDRs y su subministro
- [ ] Cobertura: falta de centros o servicios cerca de los pacientes (por ejemplo, insuficientes centros de salud en áreas rurales que realizan PDR o falta de personal sanitario en la comunidad)
- [ ] Dificultades con el manejo de casos por parte del personal sanitario (por ejemplo, el personal sanitario no sigue las guías - fallo en la realización de pruebas en todos los casos sospechosos, falta de aceptación de los resultados de la prueba de malaria, falta de conocimiento para manejar casos negativos por la PDR)
- [ ] La aceptación de la realización de pruebas y de los resultado por parte del paciente es muy baja
- [ ] Muchos pacientes buscan cuidados de proveedores que no ofrecen realizar pruebas (por ejemplo, sector privado minorista, cuidados médicos alternativos tales como remedios botánicos, curanderos)
- [ ] Desafíos relacionados con la financiación para realizar pruebas (suministros, PDR, capacitación, calidad, etc.)
- [ ] Desafíos relacionados con la realización de pruebas basadas en muestras sanguíneas (por ejemplo, manejo seguro de la sangre y de los objetos punzantes, correcta eliminación de desechos)
- [ ] Problemas relacionados con la notificación de los resultados de las pruebas, compilación de los datos de vigilancia
- [ ] Other: ____________________

15. **3.3.4. Por favor, considere el punto de vista del PROFESIONAL SANITARIO en el sector público y privado (por ejemplo, doctores, enfermeras, personal sanitario en la comunidad, farmacéuticos). ¿En qué medida alguno de los siguientes puntos de la tabla limita el logro de los objetivos de su programa nacional para la realización de pruebas diagnósticas y el tratamiento de la malaria? Por favor, seleccione una respuesta por línea:** *

Mark only one oval per row.

	No limitante en absoluto	Algo limitante	Muy limitante
Percepción de que la realización de pruebas diagnósticas lleva demasiado tiempo, dada la carga de trabajo	◯	◯	◯
No hay suficiente personal cualificado para realizar las PDR o la microscopía	◯	◯	◯
Reticencia a la hora de manejar pacientes en función de los resultados de las pruebas (por ejemplo, expectativas del paciente para el tratamiento, no querer perder una infección peligrosa, confianza en la experiencia clínica sobre los resultados de las pruebas)	◯	◯	◯
Subministro inconsistente de PDR que impide que los especialistas clínicos realicen pruebas	◯	◯	◯
Los farmacéuticos / trabajadores de farmacias son reacios a realizar pruebas basadas en sangre	◯	◯	◯
El personal sanitario no confía en la precisión de la pruebas de diagnóstico (microscopía o PDR)	◯	◯	◯
Actitud del personal sanitario: no están preocupados sobre la calidad de la prueba o sobre el manejo de casos	◯	◯	◯
Dificultad para obtener muestras de pacientes	◯	◯	◯

16. **Other (please specify)**

17. **3.3.5. Por favor, considere ahora la perspectiva de los PACIENTES, ¿en qué medida alguno de los siguientes puntos de la tabla limita el logro de los objetivos de su programa nacional para la realización de pruebas diagnósticas y el tratamiento de la malaria? Por favor, seleccione una respuesta por línea:** *

Mark only one oval per row.

	No limitante en absoluto	Algo limitante	Muy limitante
Razones culturales, religiosas, tradicionales para evitar la extracción de sangre	◯	◯	◯
Los niños prefieren evitar la punción en el dedo para extraer sangre	◯	◯	◯
Los adultos prefieren evitar la punción en el dedo para extraer sangre	◯	◯	◯
La gente evita realizarse pruebas de diagnóstico debido a los largos tiempos de espera para obtener resultados	◯	◯	◯
Los pacientes esperan un tratamiento anti-malaria, independientemente del resultado de la prueba	◯	◯	◯
No hay suficientes centros de servicio para realizar las pruebas cerca de la población que las necesita	◯	◯	◯
Conveniencia de las pruebas: los especialistas clínicos que realizan las pruebas no están disponibles durante las horas más convenientes y/o no están a una distancia conveniente (hogar/transporte)	◯	◯	◯
El coste de realización de las pruebas se percibe como elevado, tanto los costes directos como indirectos (por ejemplo, pérdida de tiempo laboral)	◯	◯	◯
Los pacientes piensan que la sangre se extrae para realizar la prueba del SIDA y no para malaria	◯	◯	◯
Los pacientes prefieren auto-tratarse/auto-medicarse	◯	◯	◯
Debido a motivos religiosos, culturales o costumbres tradicionales, los pacientes no buscan cuidado médico de los especialistas clínicos que realizan pruebas de diagnostico	◯	◯	◯
El pacientes no confían en la precisión y calidad de la microscopía o de las PDRs	◯	◯	◯
La falta de existencias de pruebas de diagnóstico y tratamiento son comunes entre los especialistas clínicos que pueden realizar estas pruebas, por lo que los pacientes evitan estos centros de salud	◯	◯	◯

18. **Other (please specify)**

DIAGNÓSTICO DE MALARIA: OBJETIVOS Y RETOS DE SU PROGRAMA

19. **Pensando en los objetivos y prioridades de su programa nacional respecto a la realización de pruebas de diagnóstico, ¿hay algún objetivo específico para el diagnóstico de la malaria o áreas de especial interés, tanto actualmente como en un futuro cercano? Por favor, describalo:**

POTENCIALES ESCENARIOS DE USO / POBLACIONES CLAVE PARA PRUEBAS NO INVASIVAS

Esta sección pretende recoger su opinión sobre el uso potencial de las pruebas de diagnóstico no invasivas y sobre las preferencias de los usuarios sobre las muestras de sangre, orina y saliva

20. **4. En su país, ¿en cuál de las siguientes situaciones podría una prueba no invasiva que usara saliva u orina ser útil?** *

Mark only one oval per row.

	Definitivamente NO es útil	Probablemente No es útil	Probablemente útil	Definitivamente útil	No aplica, el país no utilizaría este tipo de test
Realización de pruebas para el diagnóstico de malaria en los centro de salud	◯	◯	◯	◯	◯
Realización de pruebas para el diagnóstico de malaria por el personal sanitario en la comunidad	◯	◯	◯	◯	◯
Realización de pruebas para el diagnóstico de malaria en el sector privado (ej. farmacias)	◯	◯	◯	◯	◯
Detección reactiva de casos	◯	◯	◯	◯	◯
Tamizaje de poblaciones en la frontera	◯	◯	◯	◯	◯
Detección proactiva de casos en poblaciones o lugares de alto riesgo	◯	◯	◯	◯	◯
Realización de pruebas para el diagnóstico de malaria durante encuestas de población y vigilancia	◯	◯	◯	◯	◯
Auto-diagnóstico (por ejemplo, kits para realizar la prueba casa)	◯	◯	◯	◯	◯
Realización de pruebas para el diagnóstico de malaria durante brotes de enfermedades de alto riesgo (por ejemplo, ébola)	◯	◯	◯	◯	◯
Realización de pruebas para el diagnóstico de malaria en mujeres embarazadas	◯	◯	◯	◯	◯

	Definitivamente NO es útil	Probablemente No es útil	Probablemente útil	Definitivamente útil	No aplica, el país no utilizaría este tipo de test
Realización de pruebas para el diagnóstico de malaria en niños	⬭	⬭	⬭	⬭	⬭

21. **Other (please specify)**

____

22. **4.1. Si seleccionó la opción de auto-diagnóstico como probablemente o definitivamente útill, por favor, describa que grupos poblacionales y áreas se beneficiarían de esta modalidad. population groups and settings would benefit from self-testing?**

____
____
____
____
____

23. **5. Por favor, intente predecir cómo las siguientes poblaciones reaccionarían a una prueba de diagnóstico de malaria basada en SALIVA si existiera hoy en día:** *
Mark only one oval per row.

	Aceptaría fácilmente la prueba	Puede dudar en cambiar a la prueba de saliva al principio, pero eventualmente aceptaría	Prefiere la prueba basada en sangre, es improbable que use la prueba de saliva	No aplica en nuestro país
Niños	⬭	⬭	⬭	⬭
Adultos	⬭	⬭	⬭	⬭
Inmigrantes/poblaciones móviles	⬭	⬭	⬭	⬭
Indígenas	⬭	⬭	⬭	⬭
Comunidades remotas/rurales	⬭	⬭	⬭	⬭
Poblaciones evaluadas como parte de encuestas poblacionales (la mayoría sin síntomas) para vigilancia de la enfermedad	⬭	⬭	⬭	⬭
Profesionales de la salud (ej. doctores, enfermeras)	⬭	⬭	⬭	⬭
Personal sanitario de la comunidad	⬭	⬭	⬭	⬭
Minoristas (farmacias)	⬭	⬭	⬭	⬭

24. **6. Por favor, intente predecir cómo las siguientes poblaciones reaccionarían a una prueba de diagnóstico de malaria basada en ORINA si existiera hoy en día** *

Mark only one oval per row.

	Aceptaría fácilmente la prueba	Puede dudar en cambiar a la prueba de orina al principio, pero eventualmente aceptaría	Prefiere la prueba basada en sangre, es improbable que use la prueba de orina	No aplica en nuestro país
Niños	◯	◯	◯	◯
Adultos	◯	◯	◯	◯
Mujeres embarazadas	◯	◯	◯	◯
Inmigrantes/poblaciones móviles	◯	◯	◯	◯
Indígenas	◯	◯	◯	◯
Comunidades remotas/rurales	◯	◯	◯	◯
Poblaciones evaluadas como parte de encuestas poblacionales (la mayoría sin síntomas) para vigilancia de la enfermedad	◯	◯	◯	◯
Profesionales de la salud (por ejemplo. doctores, enfermeras)	◯	◯	◯	◯
Personal sanitario de la comunidad	◯	◯	◯	◯
Minoristas (farmacias)	◯	◯	◯	◯

25. **7. Por favor, comparta cualquier información relacionada con la creencias culturales sobre las extracciones de sangre o las expectativas de los pacientes sobre las pruebas de diagnóstico que utilizan sangre y sobre la malaria en su país, y como estos podrían afectar las preferencias de los pacientes sobre las pruebas de diagnóstico basadas en sangre o la pruebas no invasivas.**

POTENCIALES ESCENARIOS DE USO / POBLACIONES CLAVE PARA PRUEBAS NO INVASIVAS

26. **8. Asumiendo que funcionan igual de bien, ¿qué tipo de muestra para una prueba de diagnóstico de malaria sería la PRIMERA elección de su programa?** *

Mark only one oval.

◯ Orina
◯ Saliva
◯ Sangre

27. **9. Asumiendo que funcionan igual de bien, ¿qué tipo de muestra para una prueba de diagnóstico de malaria sería la SEGUNDA elección de su programa?** *

Mark only one oval.

- Orina
- Saliva
- Sangre

28. **10. Por favor, considere el impacto en salud pública de una prueba de diagnóstico no invasiva en su país e indique en qué grado está de acuerdo con las siguientes afirmaciones:** *

Mark only one oval per row.

	Muy en desacuerdo	En desacuerdo	De acuerdo	Muy de acuerdo	No aplica en nuestro país
Las pruebas de diagnóstico para la malaria ya son mínimamente invasivas, bien aceptadas y comunes en nuestro país, no hay necesidad de cambiarlas por pruebas no invasivas a no ser que estas tengan otras ventajas (por ejemplo, un rendimiento significativamente mejorado, menor tiempo de espera para obtener los resultados, etc.)	⬭	⬭	⬭	⬭	⬭
Las pruebas de diagnóstico no invasivas podrían aumentar significativamente el acceso a la realización de pruebas en esas poblaciones que cuestan alcanzar	⬭	⬭	⬭	⬭	⬭
En términos generales, las pruebas de diagnóstico no invasivas podrían aumentar el acceso a la realización de pruebas en nuestro país	⬭	⬭	⬭	⬭	⬭
Aumentar el número de personas que pueden realizar las pruebas es importante para nuestro programa	⬭	⬭	⬭	⬭	⬭
Las pruebas de diagnóstico no invasivas podrían aumentar la realización de pruebas en el sector privado minorista	⬭	⬭	⬭	⬭	⬭
El auto-diagnóstico (por ejemplo en casa) podría permitir que los pacientes buscaran tratamiento para la malaria antes	⬭	⬭	⬭	⬭	⬭
Utilizar sangre para realizar pruebas de diagnóstico de malaria en nuestro país no genera ningún problema	⬭	⬭	⬭	⬭	⬭

PRUEBA DIAGNÓSTICA BASADA EN SALIVA: ADOPCIÓN Y PRECIO

Para esta sección, por favor asuma que una prueba basada en SALIVA es:
- Igual de sensible que una PDR de malaria convencional, sin embargo es capaz de detectar parásitos con delecciones en HRP2 y se correlaciona bien con una infección activa
- Tan portable y fácil de utilizar como una PDR para malaria (por ejemplo, es desechable, requiere un entrenamiento mínimo del operador así como un número de pasos de procesamiento mínimo y los resultados están disponibles en menos de 30 minutos)
- Puede tener tanto una como dos bandas (especies según corresponda a su país)
- Proporciona un resultado cualitativo

29. **11. Por favor, asuma que el precio no es un problema y que este tipo de prueba basada en saliva está hoy en día disponible. ¿Cómo de probable es que su programa implemente esta prueba? ***

Mark only one oval.

- Extremadamente probable *Skip to question 30.*
- Muy probable *Skip to question 30.*
- No demasiado probable *Skip to question 31.*
- Improbable *Skip to question 31.*

PRUEBA DIAGNÓSTICA BASADA EN SALIVA: ADOPCIÓN Y PRECIO

30. **11.1. ¿Hay alguna actividad nueva que su programa consideraría hacer si hubiera una prueba de saliva disponible? Por favor, seleccione aquellas actividades de la siguiente lista que su programa podría comenzar a realizar si esta prueba estuviera disponible: ***

Check all that apply.

- [ ] Realización de pruebas para malaria por parte del personal sanitario de la comunidad
- [ ] Realización de pruebas para malaria en el sector privado minorista (por ejemplo farmacias)
- [ ] Detección reactiva de casos
- [ ] Tamizaje de poblaciones en la frontera
- [ ] Detección proactiva de casos
- [ ] Encuestas poblacionales/actividades de vigilancia que impliquen realización de pruebas de diagnóstico
- [ ] Auto-diagnóstico (por ejemplo, kits para que la población se realice las pruebas en casa)
- [ ] Auto-diagnóstico limitado (por ejemplo proporcionado por el sector público para poblaciones difíciles de alcanzar, gente que vive en áreas remotas o para poblaciones durante brotes de riesgo elevado)
- [ ] Pruebas de auto-diagnóstico para viajeros
- [ ] Realización de pruebas para malaria por parte del personal sanitario durante brotes de enfermedades de alto riesgo (por ejemplo, ébola)
- [ ] Other: ______________________

Skip to question 32.

PRUEBA DIAGNÓSTICA BASADA EN SALIVA: ADOPCIÓN Y PRECIO

31. **11.2. ¿Cuál es la principal razón por la que el programa probablemente no implementaría una prueba de diagnóstico no invasiva basada en saliva? Por favor, seleccione la respuesta más indicada.** *
Mark only one oval.

- En general, estamos satisfechos con las pruebas de diagnóstico actuales (tanto microscopía como PDRs)
- En general, falta de confianza en las pruebas de diagnóstico basadas en saliva
- Es poco probable que los especialistas clínicos acepten esta prueba de diagnóstico basadas en saliva en comparación a las que utilizan sangre
- Es poco probable que los pacientes acepten esta prueba en comparación a las que utilizan sangre
- Para cualquier nueva prueba de diagnóstico de malaria, preferimos más de 2 bandas para diferenciar entre especies
- Para cualquier nueva prueba de diagnóstico de malaria, preferimos resultados cuantitativos
- Para cualquier nueva prueba de diagnóstico de malaria, preferimos que pueda detectar infecciones asintomáticas
- Creemos que una prueba de diagnóstico de malaria basada en saliva costará más que las pruebas actuales y por lo tanto, no será asequible para el programa
- Other: ____________________

PRUEBA DIAGNÓSTICA BASADA EN SALIVA: ADOPCIÓN Y PRECIO

32. **12. Por favor, indique como su programa podría utilizar la prueba de diagnóstico basada en saliva considerando los siguientes precios:** Esta pregunta pretende ayudarnos a entender la adopción y utilización a diferentes precios. Reconocemos que responder a esto es difícil, por lo que le pedimos que proporcione su mejor estimación** Por favor, seleccione una opción por fila:** *
Mark only one oval per row.

	Probablemente no la utilice	Uso muy limitado (ej. situaciones o poblaciones concretas)	Reemplazaría parcialmente algunas de las pruebas que actualmente realizamos	Reemplazaría en gran medida las PDR que utilizamos hoy	Reemplazaría en gran medida tanto las PDR como la microscopía que actualmente utilizamos
US$0,0-0,50					
US$0,51-1,00					
US$1,01-1,50					
US>$1,51					

33. **13. Por favor, ahora asuma que esta prueba de diagnóstico basada en saliva es más sensible que una PDR tradicional y es capaz de detectar infecciones sub-microscópicas. Por favor, indique como su programa podría utilizar la prueba de diagnóstico basada en saliva considerando los siguientes precios:** *

Mark only one oval per row.

	Probablemente no la utilice	Uso muy limitado (ej. situaciones o poblaciones concretas)	Reemplazaría parcialmente algunas de las pruebas que actualmente realizamos	Reemplazaría en gran medida las PDR que utilizamos hoy	Reemplazaría en gran medida tanto las PDR como la microscopía que actualmente utilizamos
US$0,51-1,00	◯	◯	◯	◯	◯
US$1,01-1,50	◯	◯	◯	◯	◯
US>$1,51	◯	◯	◯	◯	◯

PRUEBA DIAGNÓSTICA BASADA EN ORINA: ADOPCIÓN Y PRECIO

Para esta sección, por favor asuma que una prueba basada en ORINA es:
- Igual de sensible que una PDR de malaria convencional, sin embargo es capaz de detectar parásitos con delecciones de HRP2 y se correlaciona bien con una infección activa
- Tan portable y fácil de utilizar como una PDR para malaria (por ejemplo, es desechable, requiere un entrenamiento mínimo del operador así como un número de pasos de procesamiento mínimo y los resultados están disponibles en menos de 30 minutos)
- Puede tener tanto una como dos bandas (especies según corresponda a su país)
- Proporciona un resultado cualitativo

34. **14. Por favor, asuma que el precio no es un problema y que este tipo de prueba basada en orina está hoy en día disponible. ¿Cómo de probable es que su programa implemente esta prueba?** *

Mark only one oval.

◯ Extremadamente probable *Skip to question 35.*
◯ Muy probable *Skip to question 35.*
◯ No demasiado probable *Skip to question 36.*
◯ Improbable *Skip to question 36.*

PRUEBA DIAGNÓSTICA BASADA EN ORINA: ADOPCIÓN Y PRECIO

35. **14.1. ¿Hay alguna actividad nueva que su programa consideraría hacer si hubiera una prueba de orina disponible? Por favor, seleccione aquellas actividades de la siguiente lista que su programa podría comenzar a realizar si esta prueba estuviera disponible:** *

Check all that apply.

- [ ] Realización de pruebas para malaria por parte del personal sanitario de la comunidad
- [ ] Realización de pruebas para malaria en el sector privado minorista (por ejemplo farmacias)
- [ ] Detección reactiva de casos
- [ ] Tamizaje de poblaciones en la frontera
- [ ] Detección proactiva de casos
- [ ] Encuestas poblacionales/actividades de vigilancia que impliquen realización de pruebas de diagnóstico
- [ ] Auto-diagnóstico (por ejemplo, kits para que la población se realice las pruebas en casa)
- [ ] Auto-diagnóstico limitado (por ejemplo proporcionado por el sector público para poblaciones difíciles de alcanzar, gente que vive en áreas remotas o para poblaciones durante brotes de riesgo elevado)
- [ ] Pruebas de auto-diagnóstico para viajeros
- [ ] Realización de pruebas para malaria por parte del personal sanitario durante brotes de enfermedades de alto riesgo (por ejemplo, ébola)
- [ ] Other: ____________________

Skip to question 37.

PRUEBA DIAGNÓSTICA BASADA EN ORINA: ADOPCIÓN Y PRECIO

36. **14.2. ¿Cuál es la principal razón por la que el programa probablemente no implementaría una prueba de diagnóstico no invasiva basada en orina? Por favor, seleccione la respuesta más indicada** *

Mark only one oval.

- ( ) En general, estamos satisfechos con las pruebas de diagnóstico actuales
- ( ) En general, falta de confianza en las pruebas de diagnóstico basadas en orina
- ( ) Es poco probable que los especialistas clínicos acepten esta prueba de diagnóstico basadas en orina en comparación a las que utilizan sangre
- ( ) Es poco probable que los pacientes acepten esta prueba en comparación a las que utilizan sangre
- ( ) Para cualquier nueva prueba de diagnóstico de malaria, preferimos más de 2 bandas para diferenciar entre especies
- ( ) Para cualquier nueva prueba de diagnóstico de malaria, preferimos resultados cuantitativos
- ( ) Para cualquier nueva prueba de diagnóstico de malaria, preferimos que pueda detectar infecciones asintomáticas
- ( ) Creemos que una prueba de diagnóstico de malaria basada en saliva costará más que las pruebas actuales y por lo tanto, no será asequible para el programa
- ( ) Other: ____________________

PRUEBA DIAGNÓSTICA BASADA EN ORINA: ADOPCIÓN Y PRECIO

37. **15. Por favor, indique como su programa podría utilizar la prueba de diagnóstico basada en orina considerando los siguientes precios:** Esta pregunta pretende ayudarnos a entender la adopción y utilización a diferentes precios. Reconocemos que responder a esto es difícil, por lo que le pedimos que proporcione su mejor estimación** Por favor, seleccione una opción por fila:** *

Mark only one oval per row.

	Probablemente no la utilice	Uso muy limitado (ej. situaciones o poblaciones concretas)	Reemplazaría parcialmente algunas de las pruebas que actualmente realizamos	Reemplazaría en gran medida las PDR que utilizamos hoy	Reemplazaría en gran medida tanto las PDR como la microscopía que actualmente utilizamos
US$0,0-0,50					
US$0,51-1,00					
US$1,01-1,50					
US>$1,51					

38. **16. Por favor, ahora asuma que esta prueba de diagnóstico basada en ORINA es más sensible que una PDR tradicional y es capaz de detectar infecciones sub-microscópicas. Por favor, indique como su programa podría utilizar la prueba de diagnóstico basada en ORINA considerando los siguientes precios:** *

Mark only one oval per row.

	Probablemente no la utilice	Uso muy limitado (ej. situaciones o poblaciones concretas)	Reemplazaría parcialmente algunas de las pruebas que actualmente realizamos	Reemplazaría en gran medida las PDR que utilizamos hoy	Reemplazaría en gran medida tanto las PDR como la microscopía que actualmente utilizamos
US$0.51-1.00					
US$1.01-1.50					
US>$1.51					

PREFERENCIAS PARA PRUEBAS DE DIAGNÓSTICO NO INVASIVAS

39. **17. Existe una amplia gama de pruebas posibles en función de las combinaciones de especies detectadas. Por favor, asuma que cabe la posibilidad de tener un test de diagnóstico no invasivo con dos bandas. ¿Cuál sería la configuración que su programa utilizaría más? *Asuma que Pf detecta todos los parásitos P. falciparum, incluso los que presentan delección de HRP2.** *

Mark only one oval.

- Pan/Pf
- Pf/Pv
- Pan/Pv
- Pan/Pvo
- Pf/Pvo
- Other: ____________

40. **18. Por favor, indique las preferencias de su programa para las siguientes características en una nueva prueba para el diagnóstico de malaria:** *

Mark only one oval per row.

	Absolutamente esencial	Sería bueno tenerlo; óptimo	Probablemente no es necesario
Estable a 40°C	◯	◯	◯
18 meses vida útil	◯	◯	◯
24 meses vida útil	◯	◯	◯
Resultados obtenidos más rápidamente que las PDRs actuales	◯	◯	◯
Capaz de detectar infecciones sub-microscópicas	◯	◯	◯
Resultados cuantitativos	◯	◯	◯
Podría ser utilizado por personas sin entrenamiento (siguiendo instrucciones simples, por ejemplo con dibujos)	◯	◯	◯

41. **19. Esta pregunta trata sobre lectores electrónicos: un lector es un dispositivo pequeño, portable, que funciona con batería y tiene una autonomía de aproximadamente 24 horas (sin necesidad de ser recargado). Los lectores pueden realizar múltiples funciones, incluyendo: i) mejora de la sensibilidad de la prueba; ii) automatización de la captura de datos (uso de la prueba, resultados, coordenadas GPS); y iii) mejora de la calidad guiando el procesamiento de la prueba y la interpretación de los resultados. Los inconvenientes incluyen un mayor coste y posiblemente un menor acceso debido a la suma de un dispositivo. En base a esto, ¿cómo de probable sería que su programa implementara una prueba no invasiva con un lector?** *

Mark only one oval.

- ◯ Extremadamente probable
- ◯ Muy probable
- ◯ No demasiado probable
- ◯ Improbable

FIN DE LA ENCUESTA

Gracias por participar en esta encuesta. Apreciamos su tiempo y su consideración en contestar todas nuestras preguntas. Su perspectiva es inestimable para esta iniciativa y será crucial para tomar decisiones informadas sobre los próximos pasos en el desarrollo de nuevas pruebas para el diagnóstico de malaria, así como para los inversores y grupos como FIND para entender cómo podrían apoyar en general la realización de pruebas para el diagnóstico de malaria: para el manejo de casos, la vigilancia y la eliminación.

42. **Por favor, si desea realizar cualquier otro comentario escríbalo en el siguiente recuadro:**

43. **Por separado, también estamos desarrollando una breve encuesta enfocada al personal sanitario con el fin de obtener su punto de vista en cuanto a las pruebas de diagnóstico basadas en sangre, saliva y orina. Si fuera tan amable de proporcionar los nombres y direcciones de contacto de algunas de las personas que trabajan en su país dispuestas a realizar la encuesta nos sería de gran ayuda (por ejemplo personal de enfermería, de atención integrada a las enfermedades prevalentes de la infancia, personal sanitario de la comunidad, especialistas clínicos del sector privado)**

Powered by
Google Forms
